# Design, Synthesis, and Biological Evaluation of 5,8-Dimethyl Shikonin Oximes as SARS-CoV-2 M^pro^ Inhibitors

**DOI:** 10.3390/molecules30061321

**Published:** 2025-03-14

**Authors:** Jiahua Cui, Shouyan Xiang, Qijing Zhang, Shangqing Xiao, Gaoyang Yuan, Chenwu Liu, Shaoshun Li

**Affiliations:** 1 School of Pharmacy, Gannan Medical University, Ganzhou 341000, China; tc1423177159@sjtu.edu.cn (S.X.); gmuxsq@163.com (S.X.); ygy17630814755@163.com (G.Y.); 18579009691@163.com (C.L.); 2 Jiangxi Province Key Laboratory of Pharmacology of Traditional Chinese Medicine, Gannan Medical University, Ganzhou 341000, China; 3 School of Chemistry and Chemical Engineering, Shanghai Jiaotong University, Shanghai 200240, China; 4 School of Pharmacy, Shanghai Jiaotong University, Shanghai 200240, China; lianyi0810@163.com (Q.Z.); ssli@sjtu.edu.cn (S.L.)

**Keywords:** SARS-CoV-2 M^pro^, shikonin, dimethyl shikonin oximes, inhibitors, COVID-19

## Abstract

We have designed, synthesized, and characterized a small library of shikonin derivatives and demonstrated their inhibitory activity against the main protease, M^pro^, of SARS-CoV-2. One analog, 5,8-dimethyl shikonin oxime (**15**), exhibited the highest activity against SARS-CoV-2 M^pro^ with an IC_50_ value of 12.53 ± 3.59 μM. It exhibited much less toxicity as compared with the parent compound, shikonin, in both in vitro and in vivo models. Structure–activity relationship analysis indicated that the oxime moieties on the naphthalene ring and the functional groups attached to the oxygen atom on the side chain play a pivotal role in enzymatic inhibitory activity. Molecular docking results implied that the inhibitor **15** is perfectly settled in the core of the substrate-binding pocket of M^pro^ by possibly interacting with three catalytic residues, His41, Cys145, and Met165. Overall, the shikonin oxime derivative **15** deserves further investigation as an antiviral agent against SARS-CoV-2.

## 1. Introduction

The severe acute respiratory infectious disease caused by a new coronavirus (SARS-CoV-2) led to the outbreak of pneumonia (COVID-19), which has seriously endangered the world’s public health [1,2]. According to the data from WHO, by 1 December 2024, there were more than 776.97 million diagnosed COVID-19 cases and 7.07 million confirmed deaths, making it one of the deadliest pandemics in human history [3]. From December 2021, the number of cases appears to be rapidly increasing due to the rapid epidemic expansion of the SARS-CoV-2 Omicron variant. Therefore, it is an urgent need to develop highly effective drugs against the ongoing pandemic coronavirus disease [4].

The new coronavirus is a single-strand positive-sense RNA virus with high homology to SARS-CoV and MERS-CoV [5]. After entry of the coronavirus into host cells, nuclear acids were released within the host cytoplasm and the ORF1a/b region of the viral genome translated into two polyprotein precursors (pp1a and pp1ab), which were cleaved to virus structural and non-structural proteins under the action of main protease (M^pro^) [6,7]. M^pro^ was also called the 3C-like protease because its cleavage site specificity was similar to that of picornavirus 3C protease. The non-structural proteins produced by M^pro^ were involved in the synthesis of viral subgene RNA and also four structural proteins vital for the viral reproduction. Since the main protease plays a pivotal role in the life cycle of coronavirus, and there are no homologous proteins within the host cells, M^pro^ is an ideal target for antiviral drug research and development [8,9]. Although dozens of distinct vaccines have been authorized for use against COVID-19, certain variants with mutations on their spike proteins might escape from the immune responses of vaccination [10,11].

**Figure 1:**
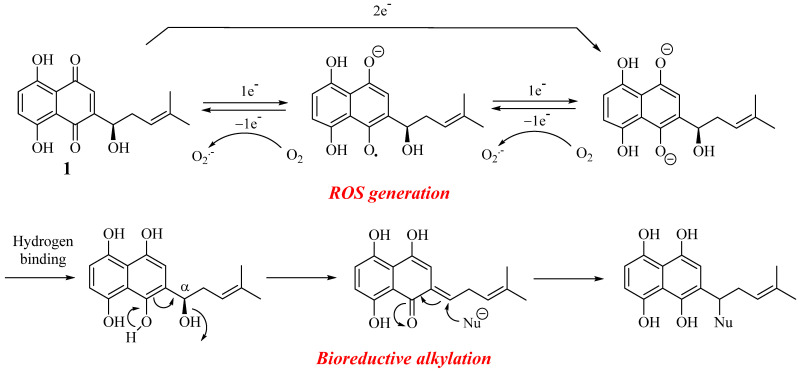
Generation of ROS from naphthazarin nucleus and bioreductive alkylation of shikonin with nucleophiles.

Natural active compounds are a wellspring of lead compounds for antiviral drug screening [12]. Shikonin (Figure 1), a natural naphthoquinone isolated from *Lithospermum erythrorhizon* Sieb. et Zucc., exhibited striking antiviral, antibacterial, anti-inflammatory, and anticancer activity. Within concentrations from 0.0156 μM to 1 μM, the naphthoquinone showed inhibitory effects against adenovirus through down-regulation of viral hexon protein and prohibition of cellular apoptosis induced by the virus [13]. Its acetyl derivative potently inhibited both infection and replication of Coxsackievirus A16 in vitro and in vivo [14]. In recent studies, it was identified as a potent inhibitor of SARS-CoV-2 M^pro^ with its IC_50_ value of 15.75 ± 8.22 μM [8].

In spite of the potent antiviral activity, it has not been used in clinical trials mainly ascribed to the non-specific cytotoxicity both in vivo and in vitro. From the chemical point of view, the naphthazarin nucleus contributed to ROS generation and bioreductive alkylation of the hallmark molecule, leading to high toxicity (Figure 1) [15]. Therefore, rational structural modifications of the naphthoquinone scaffold of **1** could prevent the cytotoxicity and also provide antiviral candidates with few side effects.

**Figure 2:**
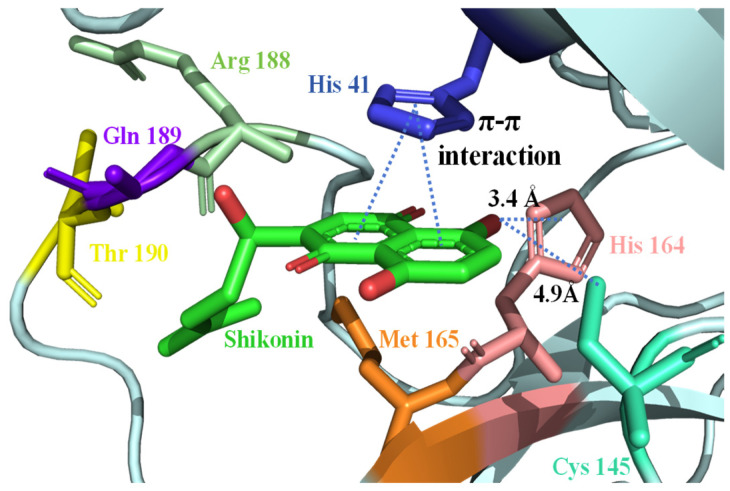
X-ray cocrystal structure of shikonin−M^pro^ complex (PDB ID: 7CA8). Shikonin in magenta color was shown in sticks. His41 (blue), His164 (yellow), and Cys145 (orange) were shown in lines.

**Figure 3:**
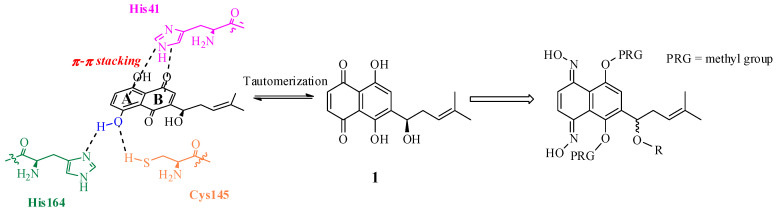
Overlooked strategy for M^pro^ inhibitor design.

The study that focused on the crystal structure of SARS-CoV-2 M^pro^ in complex with shikonin implied that the inhibitor formed multiple interactions with the target enzyme [16] (Figure 2). The phenolic hydroxyl group on the naphthazarin nucleus hydrogen bonded with protease polar triad Cys145 and His164 amino acid residues. A π–π interaction between the shikonin naphthazarin moieties with His41 on the S1 subsite also contributed to the tight binding affinity of the inhibitor with M^pro^. These findings suggested that exchange of phenolic hydroxyl groups on the A-ring of shikonin with certain hydroxyl group-contained functional groups possibly maintained the enzymatic inhibition activity. In addition, carbonyl/hydroxyl groups on the naphthazarin nucleus should be masked to avoid the generation of ROS, since ROS accumulation was correlated to the cytotoxicity of resulting compounds. The electron-withdrawing groups were not preferred since the decrease in electron density of the naphthalene ring might lead to weakened π–π interaction of prepared compounds with the side chain of His41. All of these studies motivated us to prepare 5,8-dimethyl shikonin oximes (Figure 3) and also test their enzymatic inhibition activity against SARS-CoV-2 M^pro^.

## 2. Results and Discussion

### 2.1. Synthetic Studies

**Scheme 1:**
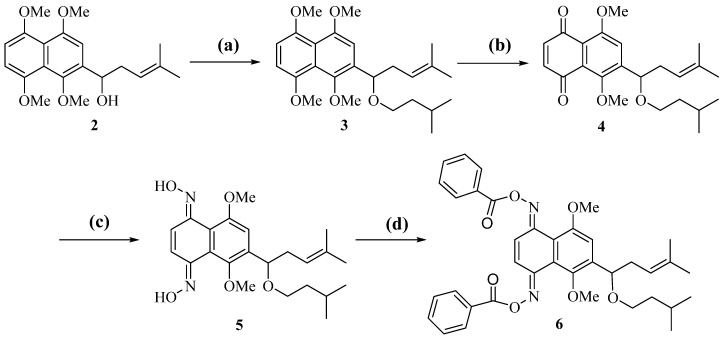
Reagents and conditions: (**a**) isoamyl bromide, NaH, KI, DMF, 0 °C, 20 min, then 60 °C, overnight; yield: 88.3%. (**b**) CAN, DCM/ACN, 0 °C, 10 min; yield: 38.7%. (**c**) NH_2_OH·HCl, Py, EtOH, 50 °C, 24 h; yield: 90.4%. (**d**) PhCOOH, EDCI, DMAP, DCM, overnight; yield: 83.2%.

Racemic 4-methyl-1-(1,4,5,8-tetramethoxynaphthalen-2-yl)pent-3-en-1-ol (**2**), which was prepared according to the reported procedures [17], was employed as the starting material in the study. The secondary alcoholic hydroxyl group of starting material was first etherified using sodium hydride and brominated isoamyl to afford compound **3** in high yield with potassium iodine as the catalyst (Scheme 1). Then, the methyl groups of **3** were cleaved by cerium ammonium nitrate (CAN)-mediated oxidation under mild conditions to afford a pair of isomers, among which naphthoquinone **4** was separated through column chromotography. Oximation of the quinone moiety of the substrate (**4**) using hydroxyl amine hydrochloride and pyridine in alcoholic solutions gave the target compound (**5**) with a high yield. Further esterification of the oxime hydroxyl group of **5** produced the ester (**6**).

**Scheme 2:**
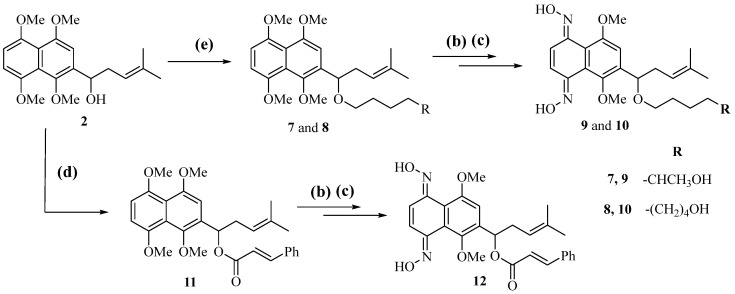
Reagents and conditions: (**e**) THP-protected bromohydrin, NaH, KI, DMF, 60 °C, overnight; then HCl, CH_3_OH, r.t., 2 h; yield for compound **7**: 81.4%; yield for compound **8**: 85.8%. (**b**)–(**d**) were the same with counterparts illustrated in Scheme 1.

Methylated shikonin analogues (**7** and **8**) were prepared by the same synthetic strategy using tetrahydropyranyl (THP) group-protected bromohydrin as a reactant instead of the brominated isopentane (Scheme 2). Acidic hydrolysis of the THP-protecting group and further oxidation–oximation reactions afforded 5,8-dimethyl shikonin oxime derivatives (**9** and **10**). Compound **12** was prepared by esterification of the secondary alcoholic hydroxyl group of **2** with cinnamoyl acid and subsequent oxidation–oximation reactions (Scheme 2).

**Scheme 3:**
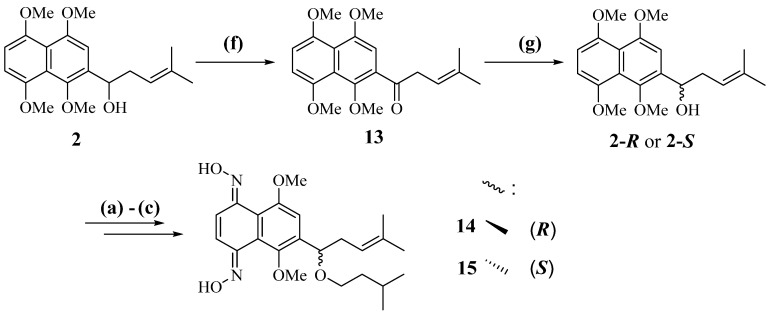
Reagents and conditions: (**f**) Dess–Martin periodinane, DCM, −15 °C, 2 h; yield: 84.5%. (**g**) (+)-DIP-Cl or (−)-DIP-Cl, −30 °C, overnight; (+)-DIP-Cl for **2-*R*** and (−)-DIP-Cl for **2-*S***. (**a**)–(**c**) were the same with counterparts illustrated in Scheme 1.

**Scheme 4:**
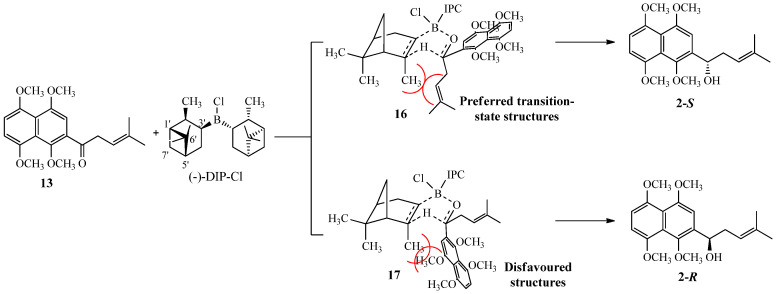
Transition-state model for (−)-DIP-Cl-mediated hydroboration of compound **13**.

In the preparation of enantiomeric secondary alcohols (**2-*R*** and **2**-***S***), the racemate **2** was first subject to moderate Dess–Martin oxidation to afford ketone **13** in high yield (Scheme 3). Further asymmetric hydrogenation of ketone **13** produced the enantiomeric alcohols **2-*R*** and **2-*S*** in excellent enantiomeric excess values using diisopinocampheyl chloroborane (+)-DIP-Cl and (−)-DIP-Cl as the reducing agent, respectively. Due to the steric hindrance of the α-pinenyl substituent, the substituted borane only hydroborates unhindered substrates, and the hydroboration reactions proceeded through the formation of six-member cyclic rings [18,19]. As shown in Scheme 4, the smaller six-carbon side chain faced the axial 2′-methyl group, while the bulky substituted naphthalene ring occupied the equatorial-like orientation in the preferred transition-state intermediate (**16**). The *cis*-elimination of boron moiety and hydrogen probably resulted in the formation of the secondary alcohol (**2-*S***) with *S*-configuration. In the disfavored intermediate (**17**), the steric repulsion between the axial 2′-methyl group and 1,4,5,8-tetramethoxy naphthalene ring prohibited the formation of *R*-enantiomer (**2**-***R***).

**Figure 4:**
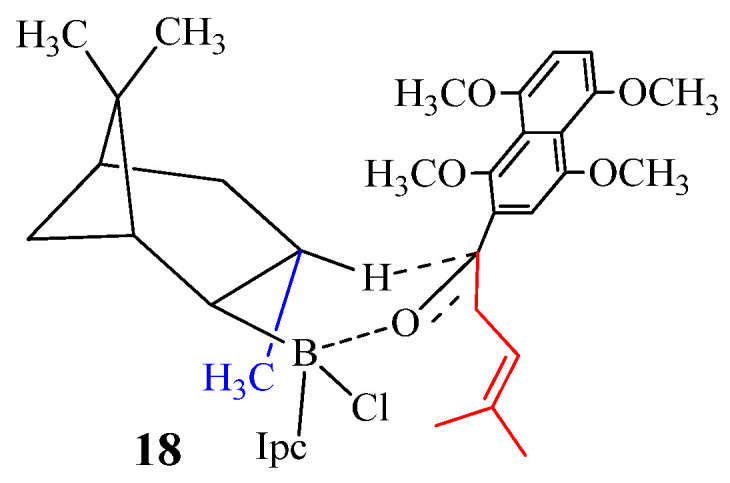
Structure of favored transition-state intermediate for (+)-DIP-Cl-mediated hydroboration.

Similarly, the reduction of ketone **13** using (+)-DIP-Cl preferentially afforded the alcohol **2-*R*** with the chiral center in *R*-configuration, and the reaction proceeded via the formation of a boat-like bicyclic transition-state intermediate (**18**, Figure 4).

**Scheme 5:**
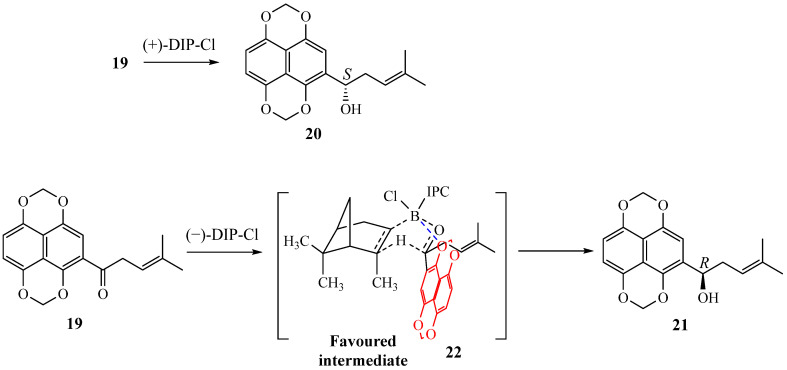
Asymmetric reduction of ketone intermediate **19** with organoborane DIP-Cl and plausible mechanisms.

The configuration of products in campheylborane DIP-Cl-mediated hydroboration of **13** as a substrate was different from the reported hydrogenation of the methylene acetal-protected analogue (**19**) using DIP-Cl [20,21] (Scheme 5). The results from previous studies indicated that the reduction of ketone intermediate **19** using (+)-DIP-Cl afforded corresponding alcohol (**20**) with the chiral center in *S*-configuration, and the (−)-DIP-Cl provided *R*-alcohol (**21**). The methoxyl group-substituted naphthalene ring of ketone **13** should be bigger in size as compared with the methylene acetal-protected naphthalene moiety of **19**. The steric repulsion between the pinocampheyl methyl group and naphthalene ring should be much weaker in intermediate **22** than that in the transition state of intermediate **17**. According to the classical theories [19,20] on DIP-Cl-mediated hydroboration reactions, there might be additional interactions between the oxygen atoms in methylene acetal-protecting groups with the boron atom, which contributed to the stabilization of transition-state intermediate **22**.

The optical purities for chiral alcohols (**2-*R*** and **2**-***S***) were determined by established chiral chromatography using the Chiralpak OD-H column with *n*-hexane-isopropanol (6:4, V/V) as the eluent [22]. The optical purity was 96% and 95% for compound **2-*R*** and **2**-***S***, respectively (see Supplementary Materials). The absolute configuration of chiral centers for two optical isomers was confirmed by comparing the chromatographic retention times of two compounds with the optical intermediate obtained by Ru(II)-catalyzed asymmetric hydrogenation [23]. In the synthesis of chiral compounds **14** and **15**, no racemization reactions were involved with **2-*R*** and **2**-***S*** as the starting material. The purity of target compounds (**14** and **15**) was confirmed by the chiral liquid chromatography with amylose tris[(S)-α-methylbenzylcarbamate] (CHIRALPAK^®^ IH, 250 × 4.6 mm, 5 µm) as the stationary phase and *n*-hexane/*i*-PrOH (9:1, V/V) as the mobile phase (compound **14**, 96% *e.e.*; compound **15**, 95% *e.e.*).

### 2.2. Enzymatic Inhibition Assay

The M^pro^ inhibition activity of prepared compounds was measured employing the reported Fluorescence Resonance Energy Transfer (FRET) method [24], in which the fluorescent MCA and Dnp were used as the donor and the acceptor, respectively. In the primary screening, compounds (**4**–**6**, **9**, **10**, and **12**) and shikonin as the positive control were tested at the concentration of 10 μM and the fluorescence intensity of each well in the enzymatic assay was monitored upon incubation for 5 min. Since racemic compound **5** was a relatively more potent inhibitor within tested 5,8-dimethyl shikonin analogues in the primary screening, compounds **14** and **15** as enantiomers and the lead shikonin were then determined for their IC_50_ values, which were in the range from 11.26 to 22.75 μM.

### 2.3. Structure–Activity Relationship Studies

The results from the preliminary enzymatic inhibition assay indicated that the substitutions on the naphthazarin moiety of shikonin and the functional groups attached to the oxygen atom on the side chain played pivotal roles in the activity (Table 1). Oximation of the quinone ring led to an increase in inhibitory activity since compound **5** was nearly a three-fold more potent inhibitor to the parent compound **4** with a quinoidal scaffold. However, benzoylation of the oxime hydroxyl group (**6**) resulted in complete loss of activity in screening. During modification of the ether linkage on the side chain, elongation of the isopentane branched chain (**5**) to an *n*-octane backbone (**10**) caused a drop in enzyme inhibition efficacy. Exchange of the isopentane group of **5** to a hexan-2-ol moiety (**9**) was also not preferred. Additionally, 5,8-dimethyl shikonin oxime (**12**) with the enzymatic inhibition rate of 28% at the concentration of 10 μM was less potent as compared with the ether derivative **5**. In the preliminary screening, the racemic compound **5** was identified as the most potent one among the tested shikonin derivatives with an enzymatic inhibition rate of about 36%.

Since most enantiomers of racemic drugs displayed marked differences in pharmacological activities [25,26], the enantiomers of compound **5** prepared through enantioselective hydrogenation were tested for their enzymatic inhibition efficacy. As shown in Table 2, *S*-enantiomer **15** was characterized as the eutomer with respect to inhibition of SARS-CoV-2 M^pro^ with the IC_50_ value of 12.53 ± 3.59 μM. It was equally potent, as compared with shikonin, as the lead (IC_50_ = 11.26 ± 2.35 μM). The eutomer **15** was a 1.8-fold more potent inhibitor as compared with the distomer (**14**). The results implied that there might be some positive or negative interactions between the isoamylene moiety on the chiral center and the isopentane substituent attached to the oxygen atom, and the combined effects influenced the M^pro^ inhibitory activity.

### 2.4. Molecular Docking Studies

**Figure 5:**
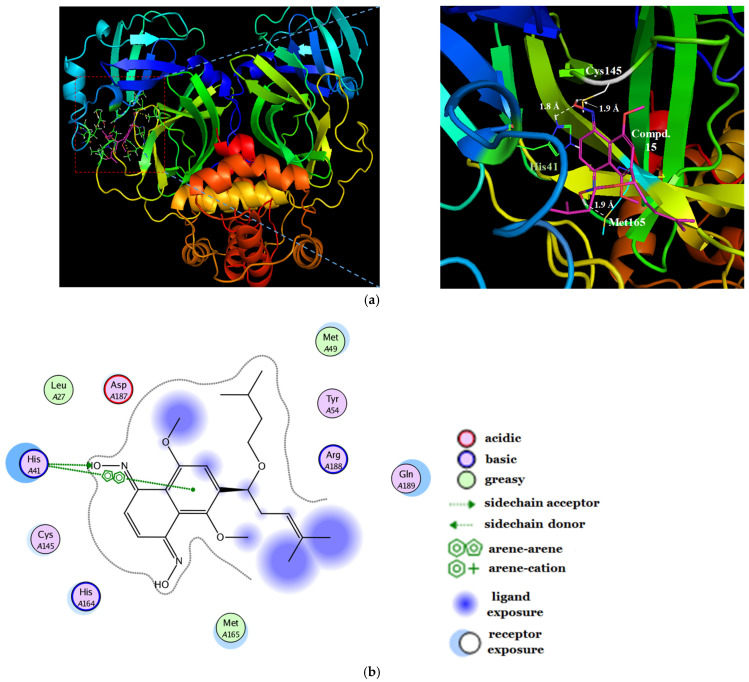
(**a**) The predicted binding model of the top-hit compound (shown as magenta sticks) in the substrate-binding pocket of SARS-CoV-2 M^pro^. White dotted lines represent hydrogen bonds with amino acid residues. (**b**) Representation of the interactions between compound **15** and surrounding amino acid residues in the SARS-CoV-2 M^pro^-binding pocket.

To further understand the putative binding modes of compound **15** against SARS-CoV-2 M^pro^, we performed a molecular docking study using the MOE 2008 software (Chemical Computing Group Inc., Montreal, QC, Canada). The X-ray crystal structure of SARS-CoV-2 M^pro^ (PDB: 7CA8) complexed with shikonin in the substrate-binding site was obtained from the RSC Protein Data Bank (Figure 5). The active site cavity was defined as amino acid residues within an 8 Å spherical radius to shikonin. In the system adaptability test of docking studies, shikonin as the native ligand was docked back to the original model (7CA8) from PDB. The result from validation studies indicated that shikonin in the predicted binding model with the lowest binding energy (−16.0859 Kcal/mol) posed a similar conformation and orientation in the active site cavity as compared with the original model. Employing the established molecular docking algorithm, compound **15** was successfully docked in the shikonin-binding pocket in SARS-CoV-2 M^pro^ with a binding energy of −16.8006 Kcal/mol. Molecular formulas, Lipinski’s rule of five, binding affinities (Kcal/mol), and binding active amino acid residues are shown in Table 3.

In the proposed binding modes (Figure 5), the inhibitor **15** was well placed in the SARS-CoV-2 M^pro^ catalytic triad region, forming hydrogen bonding with the polar Cys145 located on the S1 subsite with a distance of only 1.9 Å. In addition, the aromatic naphthalene ring in the structure of shikonin formed a π–π interaction with His41 amino acid residue on the S2 subsite. The oxygen atom on the oxime moiety also formed one hydrogen bond with the His41 imidazole functional group (bond length of 1.8 Å), contributing to the tight binding affinity. The hydrophobic interactions between the isopentene side chain and Met165 together with the hydrogen bonding between one oxime hydroxyl group and Met165 sulfur atom also confirmed the tight binding affinity of compound **15** with the substrate-binding site.

### 2.5. Surface Plasmon Resonance Assay

**Figure 6:**
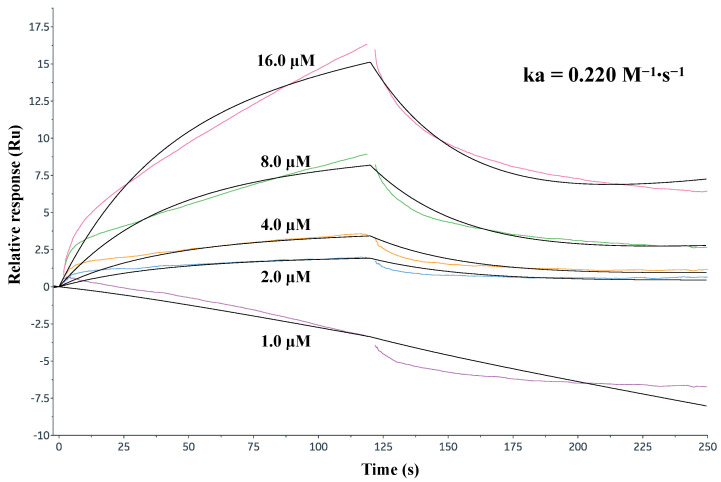
The real-time binding profile between SARS-CoV-2 M^pro^ and shikonin oxime **15**.

The real-time binding profile between SARS-CoV-2 M^pro^ protein and the inhibitor **15** was identified by the standard surface plasmon resonance (SPR) assay. SARS-CoV-2 M^pro^ proteins were immobilized to a CM5 sensorchip. For affinity analysis, compound **15** was dissolved in a PBS solution supplemented with 0.05% Tween-20 and 5% DMSO at concentrations of 1.0 μM, 2.0 μM, 4.0 μM, 8.0 μM, and 16.0 μM, respectively. We used the Biacore^TM^ insight evaluation software to calculate multi-cycle kinetics for samples. As shown in Figure 6, the binding rate constant (ka) of 0.220 M^−1^∙s^−1^ confirmed binding affinity of inhibitor **15** with the target enzyme.

### 2.6. Cytotoxicity Evaluations

Shikonin as the lead compound and 5,8-dimethyl shikonin oxime **15** were tested for their cytotoxicity using the standard MTT assay described in the Experimental Section. The results implied that shikonin was highly toxic towards the tested human normal HFF-1 and African green monkey Vero E6 cells with CC_50_ values of 1.31 ± 0.09 μM and 1.48 ± 0.06 μM (Table 4), respectively.

On the contrary, 5,8-dimethyl shikonin oxime **15** exhibited rather low cytotoxicity towards these two cell lines with CC_50_ values much higher than 50 μM. The observations were in accordance with previously published research results that the cytotoxicity of natural shikonin greatly decreased upon modifications of the naphthazarin core scaffold [28,29].

### 2.7. In Vitro Antiviral Activity

**Figure 7:**
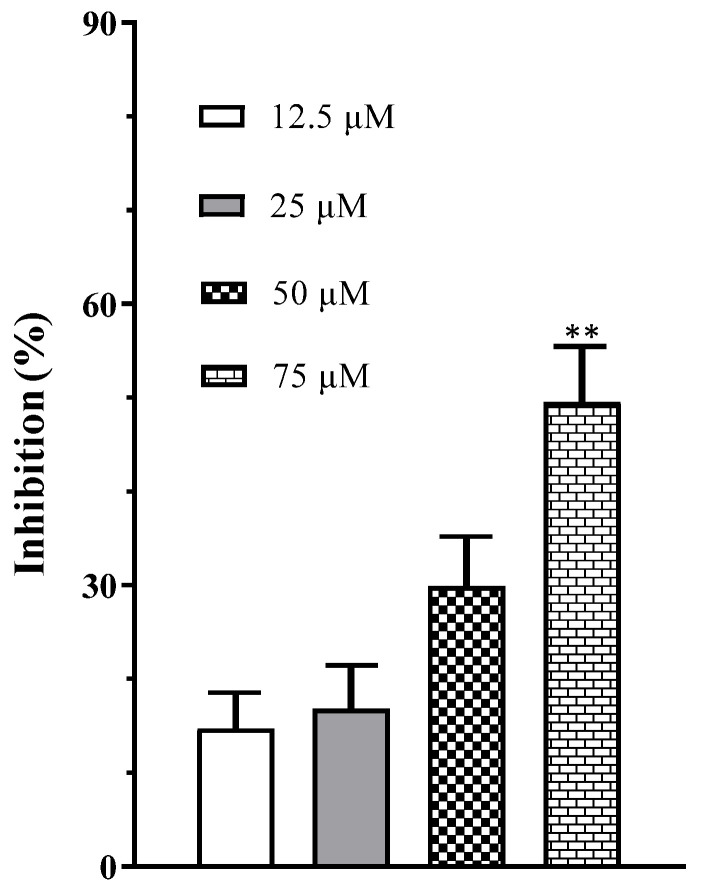
Compound **15** inhibited live SARS-CoV-2 replication in Vero E6 cells (** *p* < 0.01).

In order to determine the antiviral potential of synthesized 5,8-dimethyl shikonin oximes, we further examined the effects of compound **15** against wildtype SARS-CoV-2 replicating in Vero E6 cells. Culture supernatant was collected after treatment of the infected cells by shikonin oxime **15** at concentrations of 12.5, 25, 50, and 75 μM, respectively. Then supernatant from cell cultures was collected and SARS-CoV-2 copies were measured by qRT-PCR assay [8]. The results indicated that compound **15** at the concentration of 75 μM suppressed viral replications by about 50% in host Vero E6 cells through the quantitative RT-PCR assay (Figure 7).

**Figure 8:**
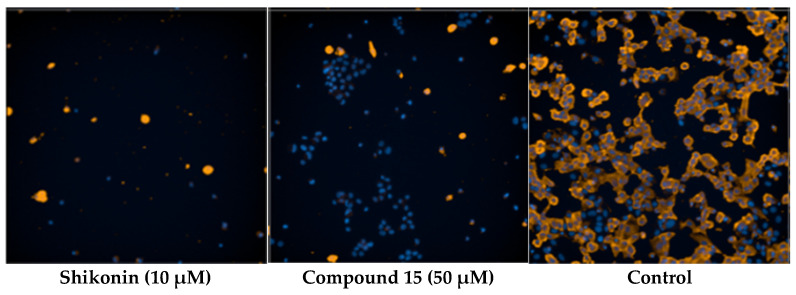
Immunofluorescent assay was used to detect the expression of SARS-CoV-2 N proteins. The yellow lights indicated viral N proteins, while the blue fluorescence from DAPI stands for nucleus of live cells.

Further immunofluorescence assays for viral N proteins were conducted to confirm the anti-viral efficacy of targeted compounds. As shown in Figure 8, cells treated with shikonin and compound **15** exhibited significantly decreased expression of viral N proteins than the untreated cells. However, due to the high toxicity of shikonin, the blue fluorescence from the live cell nucleus in the shikonin-treated group was almost invisible. All of the results implied that the shikonin oxime derivative **15** deserved further development as a potential antiviral drug candidate for the treatment of SARS-CoV-2 infections.

The therapeutic index was defined as the ratio between the antiviral EC_50_ values and the cytotoxicity. Although the shikonin analog **15** had slightly decreased enzymatic inhibitory activity than shikonin, its therapeutic index (about 0.78) was much less than that of shikonin (>1.0/0.8 ± 0.0) [30]. It deserves further investigation as an antiviral drug candidate against SARS-CoV-2.

### 2.8. In Vivo Toxicity Evaluation of 5,8-Dimethyl Shikonin Oxime 15 and Shikonin in Balb/c Mice

**Figure 9:**
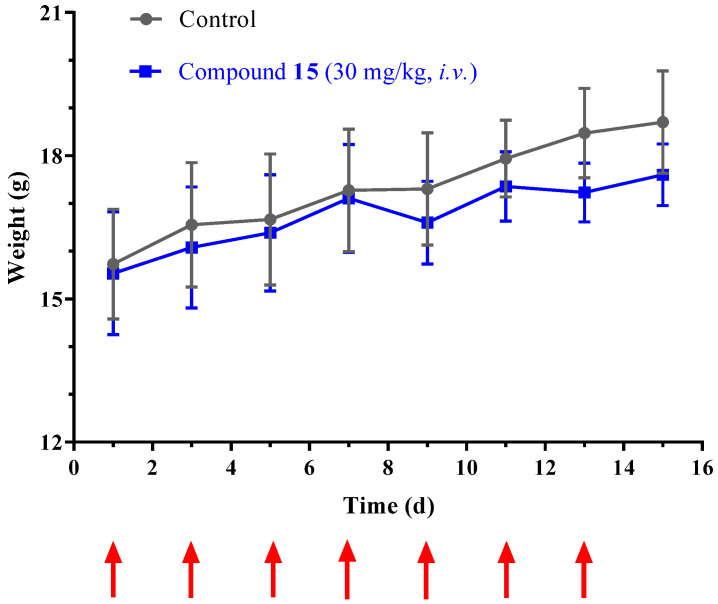
Body weight changes of animals upon treatment by compound **15**. (Arrows stands for the date of compound administration).

**Figure 10:**
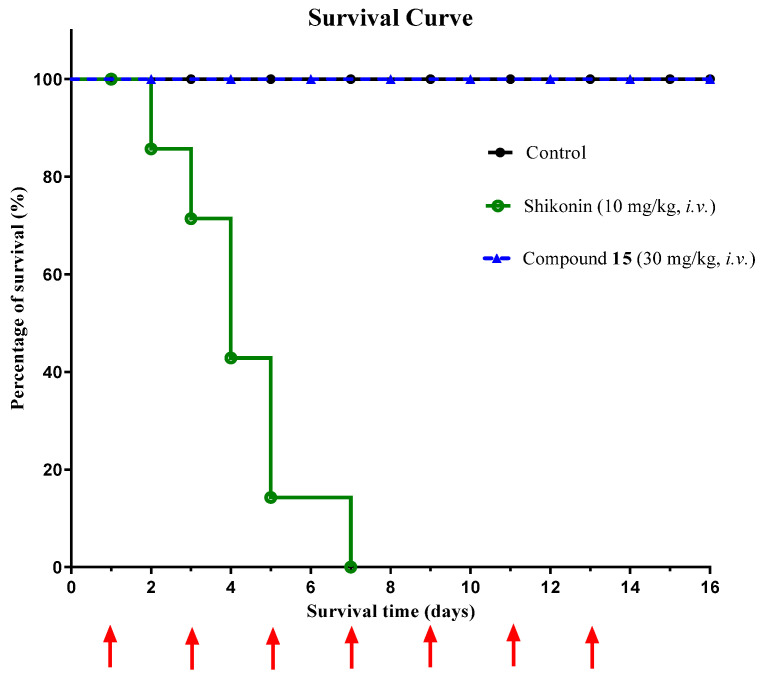
Survival curve of animals for compound **15** and shikonin group.

We performed the in vivo toxicity study of 5,8-dimethyl shikonin oxime (**15**) and lead compound shikonin in Balb/c mice by repeatedly intravenous administration of compound **15** (30 mg/kg, i.v.) (Figure 9) or shikonin (10 mg/kg, i.v.) for sive times on every other day. We monitored toxicity symptoms like loss of weight by 15% for three consecutive days, loss of appetite, slowness in activity, and treatment-related mortality, if any, from day 1 to day 13, plus two more days after the last administration. Repeated administration of compound **15** (30 mg/kg) alone did not induce any mortality or toxicity symptoms. However, the intravenous administration of shikonin (10 mg/kg) was not tolerable since all the animals died within six days after the first treatment (Figure 10). All of these observations suggested that 5,8-dimethyl shikonin oxime **15** was much less toxic in murine as compared with the parent compound shikonin, and oxime derivative **15** had certain advantages in efficacy evaluations in animals.

## 3. Experimental

### 3.1. Chemistry

Reagents and solvents in reagent grade were obtained from Shanghai Titan Scientific Co. Ltd. (Shanghai, China) and used without further purification unless otherwise stated. Melting points were determined on a WRR digital melting point apparatus (40–280 °C, Shanghai INESA Physical-Optical Instrument Co., Ltd., Shanghai, China). All NMR spectra were recorded on a Bruker Advance 400 MHz spectrometer (ideaoptics, Shanghai, China) at 400 MHz for ^1^H and 101 MHz for ^13^C or an Agilent 300 MHz spectrometer at 300 MHz for ^1^H and 75 MHz for ^13^C. All NMR measurements were carried out at room temperature and the chemical shifts are reported as parts per million (ppm) in units relative to the resonance of CDCl_3_ (7.26 ppm in the ^1^H and 77.16 ppm for the central line of the triplet in the ^13^C modes, respectively) and DMSO-*d_6_* (2.50 ppm in the ^1^H and 39.52 ppm for the central line of the septet in the ^13^C modes, respectively). The plates used for thin-layer chromatography (TLC) were E. Merck Silica Gel 60F254 (0.25 mm thickness), and they were visualized under short (254 nm) and long (365 nm) UV light. Chromatographic purifications were carried out using MN silica gel 60 (230–400 mesh). The purity of tested compounds was determined by HPLC, which was performed by using the Agilent 1100 series (Agilent Technologies Deutschland GmbH Hewlett-Packard-Strasse 8, Waldbronn, Germany) installed with an analytic column, the Agilent Zorbax C18 column (4.6 mm × 250 mm, 5 μm), at UV detection of 318 nm (reference at 450 nm) with acetonitrile (60%)/Milli-Q water (40%) at a flow rate of 1.0 mL/min. All tested compounds were shown to >98% purity according to HPLC analysis.

#### 3.1.1. Synthesis of 2-(1-(Isopentyloxy)-4-methylpent-3-en-1-yl)-1,4,5,8-tetramethoxy Naphthalene (**3**)

The 4-methyl-1-(1,4,5,8-tetramethoxynaphthalen-2-yl)pent-3-en-1-ol (**2**, 3.0 g, 8.7 mmol) was dissolved in dry DMF (30 mL) under water–ice cooling, and NaH in mineral oil (60%, 1.0 g, 25 mmol) was added in portions. After the addition, the mixture was stirred for 20 min. Then, bromoisopentane (2.6 g, 17.2 mmol) and a catalytic amount of potassium iodine were added. The reaction mixture was further headed at 60 °C overnight. After completion of the reaction, the reaction mixture was poured on ice and the mixture was extracted with dichloromethane (20 mL × 4). The combined organic layer was washed with brine, dried over anhydrous sodium sulphate, and evaporated under reduced pressure to give a brown oil, which was subjected to flash column chromatography on silica gel with 10% ethyl acetate in petroleum ether as the eluent to furnish the desired product as a light-yellow oil (3.2 g), obtaining a yield of 88.3% as well as the following: ^1^H NMR (400 MHz, CDCl_3_) δ 6.98 (s, 1H), 6.83 (s, 2H), 5.26 (t, *J* = 7.2 Hz, 1H), 4.87 (t, *J* = 6.6 Hz, 1H), 3.97–3.88 (m, 9H), 3.74 (s, 3H), 3.40–3.25 (m, 2H), 2.54–2.40 (m, 2H), 1.77–1.68 (m, 1H), 1.66 (s, 3H), 1.54 (s, 3H), 1.52–1.37 (m, 2H), 0.86 (d, *J* = 6.6 Hz, 3H), 0.81 (d, *J* = 6.6 Hz, 3H).

#### 3.1.2. Synthesis of 6-(1-(Isopentyloxy)-4-methylpent-3-en-1-yl)-5,8-dimethoxy Naphthalene-1,4-dione (**4**)

To a solution of 2-(1-(isopentyloxy)-4-methylpent-3-en-1-yl)-1,4,5,8-tetramethoxynaphthalene **3** (2.0 g, 4.8 mmol) in a mixture of dichloromethane–acetonitrile (3:1, 10 mL) under water–ice cooling, a solution of ammonium cerium nitrate (CAN, 5.8 g, 10.6 mmol) in water (8 mL) was added. After the addition, the mixture was stirred for 10 min until consumption of compound **3** as the starting material. Then, the reaction mixture was extracted with ethyl acetate (10 mL × 4), and the combined organic phase was washed with brine, dried over anhydrous sodium sulfate, and concentrated under vacuum. Finally, the residue was purified by flash chromatography on silica gel with gradient elution (20% ethyl acetate in petroleum ether to 35% ethyl acetate in petroleum ether) to furnish the desired product as a yellow-orange oil (0.72 g), with a yield of 38.7% as well as the following: ^1^H NMR (400 MHz, CDCl_3_) δ 7.72 (s, 2H), 7.22 (s, 1H), 5.26 (s, 1H), 4.77 (s, 1H), 4.04 (s, 3H), 3.71 (s, 3H), 3.39 (t, *J* = 6.6 Hz, 2H), 2.45 (s, 2H), 1.81–1.72 (m, 1H), 1.70 (s, 3H), 1.56 (s, 3H), 1.50 (q, *J* = 6.9 Hz, 2H), 0.91 (d, *J* = 6.7 Hz, 3H), 0.88 (d, *J* = 6.7 Hz, 3H).

#### 3.1.3. (1E,4E)-6-(1-(Isopentyloxy)-4-methylpent-3-en-1-yl)-5,8-dimethoxynaphthal-ene-1,4-dione Dioxime (**5**)

Hydroxylamine hydrochloride (716 mg, 10.3 mmol) and pyridine (815 mg, 10.3 mmol) were added to a solution of naphthoquinone **4** (500 mg, 1.29 mmol) in anhydrous ethanol (20 mL). The mixture was stirred at 50 °C for 24 h under nitrogen atmosphere. After completion of the reaction, solvent was evaporated under reduced pressure. Then, the residue was poured into cold water and the yellow precipitate was collected by simple filtration. The obtained filter cake was recrystallized in ethanol to afford the title compound as a light-yellow solid (487 mg), obtaining the following: yield: 90.4%; M.P. > 205 °C (decomposed); Rf value of 0.73 (50% ethyl acetate in petroleum ether); ^1^H NMR (400 MHz, DMSO-*d_6_*) δ 12.02 (s, 2H), 7.42–7.28 (m, 2H), 7.05 (s, 1H), 5.23–5.13 (m, 1H), 4.68–4.55 (m, 1H), 3.74 (s, 3H), 3.55 (s, 3H), 3.30–3.19 (m, 2H), 2.32–2.26 (m, 2H), 1.74–1.65 (m, 1H), 1.60 (s, 3H), 1.47 (s, 3H), 1.40–1.28 (m, 2H), 0.88–0.65 (m, 6H); ^13^C NMR (101 MHz, DMSO-*d_6_*) δ 153.8, 149.0, 148.0, 147.7, 138.1, 133.0, 124.4, 121.1, 120.1, 119.5, 119.4, 110.9, 75.9, 67.1, 61.2, 56.5, 38.7, 36.1, 26.0, 24.9, 23.0, 22.7, 18.1; ESI-HRMS: calcd. for C_23_H_32_N_2_NaO_5_^+^: 439.22034, found: 439.2199 [M+Na]^+^.

#### 3.1.4. (1E,4E)-6-((R)-1-(Isopentyloxy)-4-methylpent-3-en-1-yl)-5,8-dimethoxy Naphthalene-1,4-dione Dioxime (**14**)

Compound **14** as a light-yellow solid was prepared by using the above-mentioned synthetic procedures for compound **5** (Scheme 1, from ***a*** to ***c***), in which (*R*)-4-methyl-1-(1,4,5,8-tetramethoxynaphthalen-2-yl)pent-3-en-1-ol (**2-*R***) was used instead of the racemic 4-methyl-1-(1,4,5,8-tetramethoxynaphthalen-2-yl)pent-3-en-1-ol (**2**) in the etherification reaction (***a***), obtaining the following: total yield: 31.9%; M.P. > 205 °C (decomposed); Rf value of 0.73 (50% ethyl acetate in petroleum ether); ^1^H NMR (400 MHz, DMSO-*d*_6_) *δ* 12.03 (s, 2H), 7.38 (s, 2H), 7.08 (s, 1H), 5.20 (t, *J* = 7.2 Hz, 1H), 4.65 (t, *J* = 6.3 Hz, 1H), 3.78 (s, 3H), 3.59 (s, 3H), 3.31–3.29 (m, 2H), 2.39–2.29 (m, 2H), 1.79–1.66 (m, 1H), 1.64 (s, 3H), 1.51 (s, 3H), 1.46–1.32 (m, 2H), 0.86 (d, *J* = 6.6 Hz, 3H), 0.81 (d, *J* = 6.6 Hz, 3H); ^13^C NMR (101 MHz, DMSO-*d*_6_) *δ* 153.4, 148.6, 147.3, 137.7, 132.5, 124.0, 120.7, 119.7, 119.1, 110.7, 75.4, 66.7, 60.7, 56.2, 38.3, 35.6, 25.5, 24.5, 22.5, 22.3, 17.6; ESI-HRMS: calcd. for C_23_H_32_N_2_NaO_5_^+^: 439.22034, found: 439.2206 [M+Na]^+^.

#### 3.1.5. (1E,4E)-6-((S)-1-(Isopentyloxy)-4-methylpent-3-en-1-yl)-5,8-dimethoxy Naphthalene-1,4-dione Dioxime (**15**)

Compound **15** as a light-yellow solid was synthesized by using (*S*)-4-methyl-1-(1,4,5,8-tetramethoxynaphthalen-2-yl)pent-3-en-1-ol (**2-*S***) as the starting material, instead of the racemic 4-methyl-1-(1,4,5,8-tetramethoxynaphthalen-2-yl)pent-3-en-1-ol (**2**) in the synthesis, obtaining the following: total yield: 32.6%; M.P. > 205 °C (decomposed); Rf value of 0.73 (50% ethyl acetate in petroleum ether); ^1^H NMR (400 MHz, CDCl_3_) *δ* 11.86 (s, 2H), 7.77–7.63 (m, 2H), 7.20 (s, 1H), 5.30–5.22 (m, 1H), 4.77 (dd, *J* = 7.1, 5.7 Hz, 1H), 4.01 (s, 3H), 3.71 (s, 3H), 3.39 (t, *J* = 6.7 Hz, 2H), 2.45 (s, 2H), 1.81–1.71 (m, 1H), 1.70 (s, 3H), 1.56 (s, 3H), 1.50 (q, *J* = 6.7 Hz, 2H), 0.91 (d, *J* = 6.6 Hz, 3H), 0.88 (d, *J* = 6.6 Hz, 3H); ^13^C NMR (101 MHz, DMSO-*d*_6_) *δ* 153.4, 148.6, 147.6, 147.3, 137.7, 132.6, 124.0, 120.7, 119.7, 119.1, 110.6, 75.4, 66.7, 60.8, 56.2, 38.3, 35.6, 25.6, 24.5, 22.6, 22.3, 17.7; ESI-HRMS: calcd. for C_23_H_32_N_2_NaO_5_^+^: 439.22034, found: 439.2210 [M+Na]^+^.

#### 3.1.6. (1E,4E)-6-(1-((5-Hydroxyhexyl)oxy)-4-methylpent-3-en-1-yl)-5,8-dimethoxy Naphthalene-1,4-dione Dioxime (**9**)

The title compound as a yellow solid was prepared by CAN-mediated oxidation (Scheme 2, ***b***) and further oximation (***c***) with compound **7** as the starting material, obtaining the following: yield: 32.5%; M.P. > 205 °C (decomposed); Rf value of 0.38 (50% ethyl acetate in petroleum ether); ^1^H NMR (400 MHz, DMSO-*d*_6_) *δ* 11.98 (s, 2H), 7.43–7.38 (m, 2H), 7.05 (s, 1H), 5.18 (t, *J* = 6.4 Hz, 1H), 4.61 (t, *J* = 6.4 Hz, 1H), 4.23 (d, *J* = 4.6 Hz, 1H), 3.74 (s, 3H), 3.58 (s, 3H), 3.53–3.48 (m, 1H), 3.26–3.20 (m, 2H), 2.34–2.28 (m, 2H), 1.60 (s, 3H), 1.46 (s, 3H), 1.46–1.40 (m, 2H), 1.40–1.32 (m, 4H), 1.08–1.02 (m, 3H); ^13^C NMR (101 MHz, DMSO-*d*_6_) *δ* 153.9, 149.0, 148.0, 147.8, 138.2, 132.9, 124.4, 121.1, 120.1, 119.5, 111.2, 75.8, 68.9, 66.2, 61.2, 56.7, 39.2, 36.1, 30.0, 26.0, 24.0, 22.6, 18.1; ESI-HRMS: calcd. for C_24_H_34_N_2_NaO_6_^+^: 469.23091, found: 469.2315 [M+Na]^+^.

#### 3.1.7. (1E,4E)-6-(1-((8-Hydroxyoctyl)oxy)-4-methylpent-3-en-1-yl)-5,8-dimethoxy Naphthalene-1,4-dione Dioxime (**10**)

The title compound as a yellow solid was prepared by CAN-mediated oxidation (***b***) and further oximation (***c***) with compound **8** as the starting material, obtaining the following: yield: 31.6%; M.P. > 205 °C (decomposed); Rf value of 0.38 (50% ethyl acetate in petroleum ether); ^1^H NMR (400 MHz, DMSO-*d*_6_) *δ* 11.98 (s, 2H), 7.38–7.32 (m, 2H), 7.04 (s, 1H), 5.18 (t, *J* = 6.3 Hz, 1H), 4.60 (t, *J* = 6.3 Hz, 1H), 4.24–4.16 (m, 1H), 3.74 (s, 3H), 3.55 (s, 3H), 3.34–3.28 (m, 2H), 3.25–3.18 (m, 2H), 2.32–2.24 (m, 2H), 1.59 (s, 3H), 1.46 (s, 3H), 1.48–1.38 (m, 12H); ^13^C NMR (101 MHz, DMSO-*d*_6_) *δ* 153.8, 149.0, 148.0, 147.8, 138.2, 132.9, 124.4, 121.2, 120.1, 119.5, 111.1, 75.8, 68.8, 61.2, 61.2, 56.6, 36.1, 33.0, 29.8, 29.4, 29.3, 26.2, 26.0, 25.9, 18.0; ESI-HRMS: calcd. for C_26_H_38_N_2_NaO_6_^+^: 497.26221, found: 497.2621 [M+Na]^+^.

#### 3.1.8. 1-((5E,8E)-5,8-bis(Hydroxyimino)-1,4-dimethoxy-5,8-dihydronaphthalen-2-yl)-4-methylpent-3-en-1-yl Cinnamate (**12**)

4-Methyl-1-(1,4,5,8-tetramethoxynaphthalen-2-yl)pent-3-en-1-ol (**2**, 3.46 g, 10 mmol) was first esterified by cinnamic acid (2.22 g, 15 mmol), EDCI (2.88 g, 15 mmol), and DMAP (20 mg) using the same synthetic procedure for the preparation of compound **6**. Further CAN-mediated oxidation and oximation reactions furnished the title compound as a yellow solid (1.8 g), obtaining the following: total yield: 38.2%; M.P. > 188 °C (decomposed); Rf value of 0.62 (50% ethyl acetate in petroleum ether); ^1^H NMR (400 MHz, DMSO-*d*_6_) *δ* 12.08 (m, 2H), 7.89–7.61 (m, 3H), 7.54–7.26 (m, 5H), 7.14 (s, 1H), 6.73 (d, *J* = 16.0 Hz, 1H), 6.29–6.06 (m, 1H), 5.17 (s, 1H), 3.79 (s, 3H), 3.68 (s, 3H), 2.66–2.55 (m, 2H), 1.65 (s, 3H), 1.58 (s, 3H); ^13^C NMR (101 MHz, DMSO-*d*_6_) *δ* 165.61, 153.36, 147.92, 147.43, 147.20, 144.96, 135.56, 134.32, 133.96, 130.54, 128.90, 128.41, 124.15, 119.70, 119.10, 119.01, 118.01, 110.97, 70.36, 60.54, 56.47, 33.86, 25.51, 17.70; ESI-HRMS: calcd. for C_27_H_28_N_2_NaO_6_^+^: 499.18396, found: 499.1843 [M+Na]^+^.

#### 3.1.9. Synthesis of (1E,4E)-6-(1-(Isopentyloxy)-4-methylpent-3-en-1-yl)-5,8-dimethoxynaphthalene-1,4-dione O,O-Dibenzoyl Dioxime (**6**)

A mixture of 5,8-dimethyl shikonin oxime **5** (208 mg, 0.5 mmol), benzoic acid (195 mg, 1.6 mmol), EDCI (307, 1.6 mmol), and catalytic amount of DMAP (20 mg) were dissolved in dry dichloromethane, and the mixture was stirred at room temperature overnight. After completion of the reaction, the mixture was diluted with cold water and extracted by DCM (10 mL × 4). The usual work-up including washing and solvent evaporation afforded a residue, which was subject to flash chromatography with 10% ethyl acetate in petroleum ether as the eluent to furnish the ester **6** as a yellow solid (260 mg), obtaining the following: yield: 83.2%; M.P. > 230 °C (decomposed); Rf value of 0.82 (50% ethyl acetate in petroleum ether); ^1^H NMR (400 MHz, CDCl_3_) *δ* 8.16–8.12 (m, 2H), 8.11 (dd, *J* = 2.9, 1.4 Hz, 2H), 7.71–7.63 (m, 2H), 7.65–7.59 (m, 2H), 7.54–7.48 (m, 4H), 7.25 (s, 1H), 6.11 (dd, *J* = 17.6, 10.8 Hz, 1H), 4.96 (dd, *J* = 10.8, 1.4 Hz, 1H), 4.79 (dd, *J* = 17.6, 1.5 Hz, 1H), 4.52 (s, 1H), 4.01 (s, 3H), 3.99 (s, 3H), 3.34–3.28 (m, 2H), 1.77–1.71 (m, 1H), 1.46–1.40 (m, 2H), 1.14 (s, 3H), 0.94 (s, 3H), 0.90 (s, 3H), 0.85 (s, 3H); ^13^C NMR (101 MHz, CDCl_3_) *δ* 163.3, 163.2, 154.2, 153.9, 153.4, 151.6, 144.5, 139.3, 133.6, 133.5, 129.7, 129.7, 128.8, 128.7, 128.7, 128.7, 123.3, 122.8, 122.8, 117.6, 115.9, 112.4, 82.1, 68.1, 62.1, 56.9, 42.6, 38.8, 25.2, 25.0, 22.7, 22.6; ESI-HRMS: calcd. for C_37_H_40_N_2_NaO_7_^+^: 647.27277, found: 647.2732 [M+Na]^+^.

#### 3.1.10. Synthesis of 6-((4-Methyl-1-(1,4,5,8-tetramethoxynaphthalen-2-yl)pent-3-en-1-yl)oxy)hexan-2-ol (**7**) and 8-((4-Methyl-1-(1,4,5,8-tetramethoxynaphthalen-2-yl)pent-3-en-1-yl)oxy)octan-1-ol (**8**)

4-Methyl-1-(1,4,5,8-tetramethoxynaphthalen-2-yl)pent-3-en-1-ol (**2**, 0.5 g, 1.4 mmol) in dry DMF (15 mL) was treated with NaH (0.45 g, 60% in mineral oil, 18.8 mmol) at 5 °C. Then, the mixture was stirred for 20 min and 2-((6-bromohexan-2-yl)oxy)tetrahydro-2H-pyran (0.74 g, 2.8 mmol) and potassium iodine (46 mg) were added. The reaction mixture was heated at 60 °C for 12 h and poured on ice after consumption of compound **2** as the starting material. The usual extraction work-up using dichloromethane and further concentration afforded a yellow oil, which was purified by flash column chromatography on silica gel with 10% ethyl acetate in petroleum ether as the eluent to furnish the THP protected intermediate (0.68 g). The intermediate was digested in a mixture of concentrated hydrochloric acid–methanol (1:10, V/V, 11 mL) and the resulting solution was stirred for 2 h at room temperature. Then, the solution was diluted with dichloromethane (10 mL) and carefully neutralized by a saturated sodium bicarbonate solution. The separated organic layer was separated, washed with brine, dried over sodium sulphate anhydrous, and concentrated. The residue was purified by flash chromatography on silica gel with gradient elution (20% ethyl acetate in petroleum ether to 35% ethyl acetate in petroleum ether) to furnish the desired product as a light-yellow oil (0.51 g), obtaining the following: yield: 81.4%; ^1^H NMR (400 MHz, CDCl_3_) *δ* 6.92 (s, 1H), 6.78–6.74 (m, 2H), 5.22–5.18 (m, 1H), 4.85–4.81 (m, 1H), 4.03–4.00 (m, 1H), 3.94–3.61 (m, 12H), 3.32–3.18 (m, 2H), 2.46–2.40 (m, 2H), 2.03–1.96 (m, 1H), 1.62 (s, 3H), 1.48 (s, 3H), 1.45–1.11 (m, 6H), 1.10–1.02 (m, 3H); ^13^C NMR (101 MHz, CDCl_3_) *δ* 153.4, 151.4, 150.2, 147.4, 133.1, 132.7, 122.4, 120.7, 108.2, 107.6, 105.8, 75.2, 68.7, 67.7, 62.7, 57.7, 57.0, 56.9, 56.8, 39.0, 36.1, 29.7, 25.7, 23.3, 22.5, 17.9.

Compound **8** was prepared using 2-((8-bromooctyl)oxy)tetrahydro-2H-pyran instead of 2-((6-bromohexan-2-yl)oxy)tetrahydro-2H-pyran as the starting material in the synthetic procedure for compound **7** (Scheme 2), obtaining the following: yield: 85.8%; ^1^H NMR (400 MHz, CDCl_3_) *δ* 6.89 (s, 1H), 6.72–6.66 (m, 2H), 5.21–5.15 (m, 1H), 4.82–4.74 (m, 1H), 3.82 (s, 3H), 3.79 (s, 3H), 3.75 (s, 3H), 3.63 (s, 3H), 3.46–3.40 (m, 2H), 3.25–3.16 (m, 2H), 2.80–2.71 (m, 1H), 2.44–2.35 (m, 2H), 1.55 (s, 3H), 1.43 (s, 3H), 1.40–1.34 (m, 2H), 1.31–1.08 (m, 10H); ^13^C NMR (101 MHz, CDCl_3_) *δ* 153.3, 151.3, 150.1, 147.3, 132.9, 132.7, 122.4, 120.8, 120.1, 108.2, 107.6, 105.8, 75.2, 68.8, 62.5, 62.4, 57.6, 56.8, 56.8, 36.1, 32.6, 29.8, 29.3, 29.2, 26.1, 25.6, 20.8, 17.8.

#### 3.1.11. Synthesis of 4-Methyl-1-(1,4,5,8-tetramethoxynaphthalen-2-yl)pent-3-en-1-one (**13**)

2-(1-Hydroxy-4-methyl-3-pentenyl)-1,4,5,8-tetramethoxynaphthalene (**2**, 5.0 g, 14.4 mmol) was dissolved in dry CH_2_Cl_2_ (50 mL) cooled in a salt-ice bath at −15 °C. Dess–Martin periodinane (DMP, 7.3 g, 17.3 mmol) was added in portions, and the mixture was stirred for 2 h. After the consumption of the substrate, the excessive oxidant was removed by filtration, and the filtrate was concentrated under reduced pressure. The residue was purified by column chromatography with 20% ethyl acetate in petroleum ether as the eluent to obtain 4.2 g of the ketone **13** as a pale-yellow oil, obtaining the following: yield: 84.5%; ^1^H NMR (400 MHz, CDCl_3_) *δ* 6.83 (d, *J* = 2.3 Hz, 1H), 6.72–6.64 (m, 2H), 5.38–5.30 (m, 1H), 3.77–3.72 (m, 6H), 3.70 (d, *J* = 6.9 Hz, 2H), 3.67 (s, 3H), 3.60 (s, 3H), 1.57 (s, 3H), 1.48 (s, 3H); ^13^C NMR (101 MHz, CDCl_3_) *δ* 202.7, 153.1, 151.0, 150.9, 150.0, 134.8, 130.3, 122.3, 122.1, 116.8, 110.5, 108.0, 105.6, 63.7, 57.5, 56.6, 56.5, 42.8, 25.6, 18.0.

#### 3.1.12. Synthesis of (S)-4-Methyl-1-(1,4,5,8-tetramethoxynaphthalen-2-yl)pent-3-en-1-ol (**2-S**) and (R)-4-Methyl-1-(1,4,5,8-tetramethoxynaphthalen-2-yl)pent-3-en-1-ol (**2-R**)

The 4-methyl-1-(1,4,5,8-tetramethoxynaphthalen-2-yl)pent-3-en-1-one (**13**, 5.0 g, 14.4 mmol) was dissolved in anhydrous THF (60 mL) and the solution was stirred for 5 min at −30 °C temperature under nitrogen atmosphere. Then, (−)-DIP-Cl (1.7 M in *n*-hexane, 12.8 mL, 21.8 mmol) was dropwise added to the solution and the mixture was maintained at −30 °C overnight. After the completion of reaction, a few drops of cold acetone were added. The quenched reaction mixture was poured into a saturated NH_4_Cl solution and extracted with ethyl acetate (30 mL × 5). The organic layer was washed with brine, dried over anhydrous sodium sulphate, and concentrated under reduced pressure. The residue was purified by column chromatography with 35% ethyl acetate in petroleum ether as the eluent to obtain compound (**2-*S***) as a yellow oil (4.5g), obtaining the following: yield: 89.5%; ^1^H NMR (300 MHz, CDCl_3_) *δ* 7.02 (s, 1H), 6.82 (s, 2H), 5.32–5.19 (m, 2H), 3.95 (s, 3H), 3.93 (s, 3H), 3.89 (s, 3H), 3.77 (s, 3H), 2.53 (t, *J* = 7.0 Hz, 2H), 2.32 (s, br, 1H), 1.73 (s, 3H), 1.65 (s, 3H).

Compound (**2-*R***) as a yellow oil was prepared by asymmetric hydrogenation of 4-methyl-1-(1,4,5,8-tetramethoxynaphthalen-2-yl)pent-3-en-1-one (**13**) using (+)-DIP-Cl, obtaining the following: yield: 87.3%; ^1^H NMR (400 MHz, CDCl_3_) δ 7.02 (s, 1H), 6.82 (s, 2H), 5.25 (q, *J* = 7.7, 7.0 Hz, 2H), 3.95 (s, 3H), 3.94 (s, 3H), 3.90 (s, 3H), 3.77 (s, 3H), 2.53 (t, *J* = 7.1 Hz, 2H), 2.33 (s, br, 1H), 1.73 (s, 3H), 1.65 (s, 3H).

### 3.2. Biological Activity Evaluations

Methyl thiazolyl tetrazolium (MTT) was purchased from Sigma-Aldrich. DMEM, fetal bovine serum (FBS), and penicillin/straptomycin were obtained from Hyclone. The recombinant SARS-CoV-2 M^pro^ was expressed and purified from an *Escherichia coli* expression system [8]. Shikonin (purity in HPLC > 98%, *e.e.* value of 96%) as the positive control in the enzymatic assay was synthesized by the reported procedures [23].

#### 3.2.1. Enzymatic Inhibition Assay

The inhibitory potency of the tested naphthoquinones against SARS-CoV-2 M^pro^ was evaluated according to the reported FRET detection method [8,12], using fluorescently labeled peptide MCA-AVLQSGFR-Lys(Dnp)-Lys-NH_2_ as the substrate. Upon incubation, the fluorescence intensity of each well was determined by a FlexStation3 microplate reader (Molecular Device, Silicon Valley, CA, USA) with the excitation and emission filter set at 320 and 405 nm, respectively.

#### 3.2.2. Molecular Docking

Molecular docking studies were carried out using MOE 2008 software and taking SARS-CoV-2 M^pro^ as the target enzyme. The protein was downloaded from the RSC Protein Data Bank (PDB ID: 7CA8). Before docking studies, ligands and water molecules are removed from the downloaded protein, and then gasteiger charges are calculated after addition of polar hydrogens. The chemical structures of shikonin and compound **15** was generated by ChemDraw Ultra 12.0 software and structural energy minimization was conducted using Chem3D Pro 12.0. For docking, we used default values of parameters in MOE 2008, except for the first scoring function, where ASE scoring was used instead of the defaulting London dG. The type of post-placement refinement and the rescoring algorithm were set as forcefield and ASE, respectively. Then, the obtained optimal binding modes of shikonin and compound **15** in active site cavities of SARS-CoV-2 M^pro^ were employed to evaluate the reasonability of the predicted interactions.

#### 3.2.3. Surface Plasmon Resonance Analysis

The surface plasmon resonance (SPR)-based measurements were performed by the Biacore 8K apparatus. Briefly, the SARS-CoV-2 M^pro^ proteins were immobilized to an activated CM5 sensorchip (Cytiva) using an amino group coupling kit. For affinity analysis, the inhibitor was dissolved in a PBS buffered solution (PBS-T) containing 0.05% Tween-20 (V/V) and 5% DMSO (V/V) at final concentrations of 1.0 μM, 2.0 μM, 4.0 μM, 8.0 μM, and 16.0 μM. Each sample that was bound to the target enzyme was dissociated by the PBS-T buffer solution for 120 s at a flow rate of 20 μL/min. Regeneration of sensor chips was performed for 30 s using a regeneration buffer solution (pH 2.0). The binding profile for each concentration and the binding rate constant (ka) were determined by the Biacore (Cytiva) apparatus.

#### 3.2.4. Cell Lines

The human HFF-1 and monkey Vero E6 cells were provided by Cell Bank in Shanghai, Chinese Academy of Sciences. HFF-1 cells were cultured in DMEM complete medium containing 15% FBS, 100 U/mL penicillin, and 100 μg/mL of streptomycin, while Vero E6 cells were maintained in DMEM complete medium containing 10% FBS. They were maintained at 37 °C in a humidified atmosphere with 5% CO_2_ (V/V).

#### 3.2.5. Cytotoxicity Evaluation

The in vitro cytotoxicity of compounds was determined by the standard MTT assay according to our reported procedure. Briefly, well-cultured cells were seeded in 96-well plates at a density of 5 × 10^3^ cells per well. After being cultured overnight in a 37 °C humidified incubator (5% CO_2_, V/V), cells were incubated with tested compounds for 48 h. Then, cells in each well were treated with MTT in PBS (5 mg/mL) for 4 h at 37 °C, and formazan crystals in viable cells were dissolved by dimethyl sulfoxide (150 μL) at ambient temperature. The absorbance of solubilized formazan was measured by the Synergy multimode reader (BioTek, Winooski, VT, American) at the wavelength of 490 nm.

#### 3.2.6. In Vitro Antiviral Activity Evaluation

The in vitro antiviral activity of compound **15** at concentrations of 12.5 μM, 25 μM, 50 μM, and 75 μM against SARS-CoV-2 was evaluated according to the reported qRT-PCR assay [8]. To further confirm the anti-SARS-CoV-2 activity of the target compound, we perform more anti-viral assays through viral N-protein detection. Human Vero E6 cells were infected by live SARS-CoV-2 at an MOI value of 0.01. Cells were then washed 3 times with PBS to remove the free virus after 2 h-infection and then maintained in DMEM culture medium containing compound **15** (50 μM) or shikonin (10 μM) and 2% FBS for 48 h. Then, cells in each group were collected and the immunofluorescence assay was used to detect the expression of viral N protein, and the result was analyzed by high-content screening.

#### 3.2.7. In Vivo Toxicity Evaluation of Compound 15 in Balb/c Mice

Five- to six-week-old female Balb/c mice, weighing between 14 and 16 g, were obtained from Charles River Laboratories in China. After arrival, these Balb/c mice were quarantined for 3 days before housing in a germ-free environment with a 12 h-of-light and 12 h-of-dark cycle. All investigation procedures were performed according to the regulation of the Animals Ethics Subcommittee of Shanghai Jiao Tong University (A2019113).

The stock solution of compound **15** (9 mg/mL) for in vivo experiments was prepared by dissolving the powder of compound **15** (90 mg) in N-methyl-2-pyrrolidone (NMP, 5.0 mL), followed by addition of Cremophor EL in an equal volume (5.0 mL). The stock solution was diluted by sterilized saline to afford compound **15** formulation (1.8 mg/mL). In the formulation, the ratio of NMP, Cremophor EL, and saline was 1:1:8. The stock solution of shikonin (6 mg/mL) for in vivo experiments was prepared by dissolving shikonin (98%, 60 mg) in a mixture of ethanol–Cremophor EL (1:1, V/V, 10 mL). The formulation (1 mg/mL) was obtained by dilution of the stock solution with a 5-fold volume of saline.

In the in vivo toxicity evaluations, six-week-old female Balb/c mice were randomized into 3 groups, with 7 mice per group. Groups 1–3 were as follows: (**1**) compound **15** solvent; (**2**) compound **15** (30 mg/kg); (**3**) shikonin (10 mg/kg). In addition, 5,8-dimethyl shikonin oxime **15** was intravenously administrated (i.v.) on every other day for a consecutive 14 days. Administration of shikonin (i.v.) was also conducted on every other day for 7 times from day 1 to day 14. During the treatment, the body weight and activity of mice were monitored. Low activity, body weight loss of more than 15% for 3 consecutive days, or treatment-related mortality were regarded as toxicity symptoms. Mice were observed for 2 more days after the end of treatment for any delayed toxicity.

## 4. Conclusions and Future Perspectives

The research results from previous studies indicated that the natural bioactive compound shikonin was a strong inhibitor of SARS-CoV-2 M^pro^ [8,16]. Cross-validation studies have shown that shikonin only inhibited M^pro^ in the absence of a reducing reagent such as DTT, suggesting that shikonin is a non-specific cysteine protease inhibitor, leading to cytotoxicity towards host cells. In addition, the generation of reactive oxygen species (ROS) and bioreductive alkylation reactions with biomolecules were considered as the roots for its pervasive toxicity. Apparently, modification of the shikonin naphthazarin scaffold might provide a good template for the discovery of viral M^pro^ inhibitors.

We have designed, synthesized, and characterized a small library of shikonin derivatives and demonstrated that they are inhibitory in vitro against the main protease of SARS-CoV-2 (M^pro^), which causes COVID-19, a serious disease epidemic in 280 countries worldwide. Compound **15** as the most active one showed an IC_50_ value of 12.53 ± 3.59 µM against SARS-CoV-2 M^pro^ as a key antiviral drug target. The results of molecular docking studies indicated that inhibitor **15** is perfectly settled in the core of the substrate-binding pocket of M^pro^ by possibly interacting with three catalytic residues, His41, Cys145, and Met165. The target compound **15** exhibited much less toxicity as compared with the lead shikonin both in vivo and in vitro. The antiviral activity of the compound was assessed against SARS-CoV-2 where it exhibited strong inhibition at the concentration of 75 µM. The unique chemical structure and its strong enzymatic inhibition potency, coupled with the favorable safety data both in vivo and in vitro, emphasized that the shikonin oxime derivative **15** deserves further development as an antiviral drug candidate against SARS-CoV-2 in the future. Additionally, taking compound **15** as a new lead compound, we would possibly obtain more safe and effective antiviral agents to combat infection caused by SARS-CoV-2.

## Figures and Tables

**Table 1:** Enzymatic inhibition rates of tested compounds in the primary screening.

No. of Compd.	Enzymatic Inhibition Rate (%)
**4**	10.6
**5**	35.8
**6**	−6.9
**9**	10.7
**10**	11.3
**12**	28.0
**1**	51.4

**Table 2:** IC_50_ values of selected enantiomers against SARS-CoV-2 M^pro^.

No. of Compd.	Chemical Structure	IC_50_ (μM)
**14**	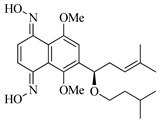	22.75 ± 4.61
**15**	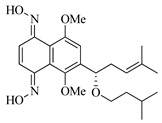	12.53 ± 3.59
**1**	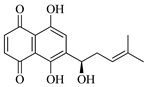	11.26 ± 2.35

**Table 3:** Properties, Lipinski’s rule of five, binding affinities, and active amino acid residues in docking studies.

Compd.	Molecular Formula	Lipinski’s Rule of Five		Calculated Binding Energy (Kcal/mol)	Binding Amino Acid Residues
**15**	C_23_H_32_N_2_O_5_	Molecular weight (<500 DA)	416.51	−16.8006	**His41**, **Cys145**, **His164**, **Met165**, **Thr190**, **Arg188**, **Gln189**
		LogP (<5)	4.29 *		
		H-bond donor (5)	2		
		H-Bond acceptor (10)	7		
Shikonin	C_16_H_16_O_5_	Molecular weight (<500 DA)	288.30	−16.0859	**His41**, **Cys145**, **His164**, **Met165**, **Tyr54**, **Arg188**, Asp187, **Gln189**, Met49
		LogP (<5)	0.92 *; 1.08 ^Δ^		
		H-bond donor (5)	3		
		H-Bond acceptor (10)	5		

Note: * the data were predicted by ChemDraw Ultra 12.0 software; ^Δ^ stands for the experimental value [27].

**Table 4:** Cytotoxic activity of shikonin and compound **15** against selected mammalian cell lines.

No. of Compd.	Chemical Structures	CC_50_ Values	
		HFF-1	Vero E6
**15**	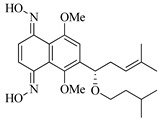	85.6 ± 5.9 μM	96.7 ± 8.3 μM
**1**	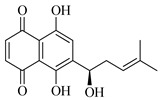	1.31 ± 0.09 μM	1.48 ± 0.06 μM

## Data Availability

The raw data supporting the conclusions of this article will be made available by the authors on request.

## Supplementary Materials

The following supporting information can be downloaded at https://www.mdpi.com/article/10.3390/molecules30061321/s1: Figures S1–S11. Representative ^1^H & ^13^C-NMR Spectra; HPLC trace for chiral separation of 2-R and 2-S as the key intermediates; HPLC trace for compound 15.
