# Supplementary material for: Design, Synthesis, and Biological Evaluation of 5,8-Dimethyl Shikonin Oximes as SARS-CoV-2 Mpro Inhibitors
Source: Molecules. 2025 Mar 14;30(6):1321. doi: 10.3390/molecules30061321 (PMC11945236; doi:10.3390/molecules30061321)
Supplement: Supplementary file 1 [file molecules-30-01321-s001.zip › molecules-3405376-supplementary.pdf]

Supplementary Information

Design, synthesis and biological evaluation of 5,8-dimethyl shikonin oximes as SARS-CoV-2 M^{pro} inhibitors

Jiahua Cui[#,* 1,2], Shouyan Xiang[# 1,3], Qijiang Zhang[4], Shangqing Xiao[1], Gaoyang Yuan[1], Chenwu Liu[1] and Shaoshun Li[4]

[1]*School of Pharmacy, Gannan Medical University, Ganzhou, Jiangxi, China*

[2]*Jiangxi Province Key Laboratory of Pharmacology of Traditional Chinese Medicine, Gannan Medical University, Ganzhou, Jiangxi, China*

[3]*School of Chemistry and Chemical Engineering, Shanghai Jiaotong University, Shanghai, China*

[4]*School of Pharmacy, Shanghai Jiaotong University, Shanghai, China*

Correspondence:

Name: Jiahua Cui

ORCID ID: 0000-0001-7164-6851

Add/Affiliation: School of Pharmacy, Gannan Medical University, Ganzhou, Jiangxi, China

Tel: +86-21-34204775

Fax: +86-21-34204775

E-mail: cpucjh@sjtu.edu.cn

#: Authors contributed equally to this work.

Table of Contents

Figures S1–S11. Representative ^{1}H & ^{13}C-NMR Spectra 3

HPLC trace for chiral separation of 2-*R* and 2-*S* as the key intermediates 14

HPLC trace for compound **15**.. 15

Figures S1–S9. Representative ^{1}H & ^{13}C-NMR Spectra

Figure S1. ^{1}H-NMR Spectrum of **2-(1-(isopentyloxy)-4-methylpent-3-en-1-yl)-1,4,5,8-tetramethoxynaphthalene (3)**

^{1}H-NMR ($CDCl_3$)

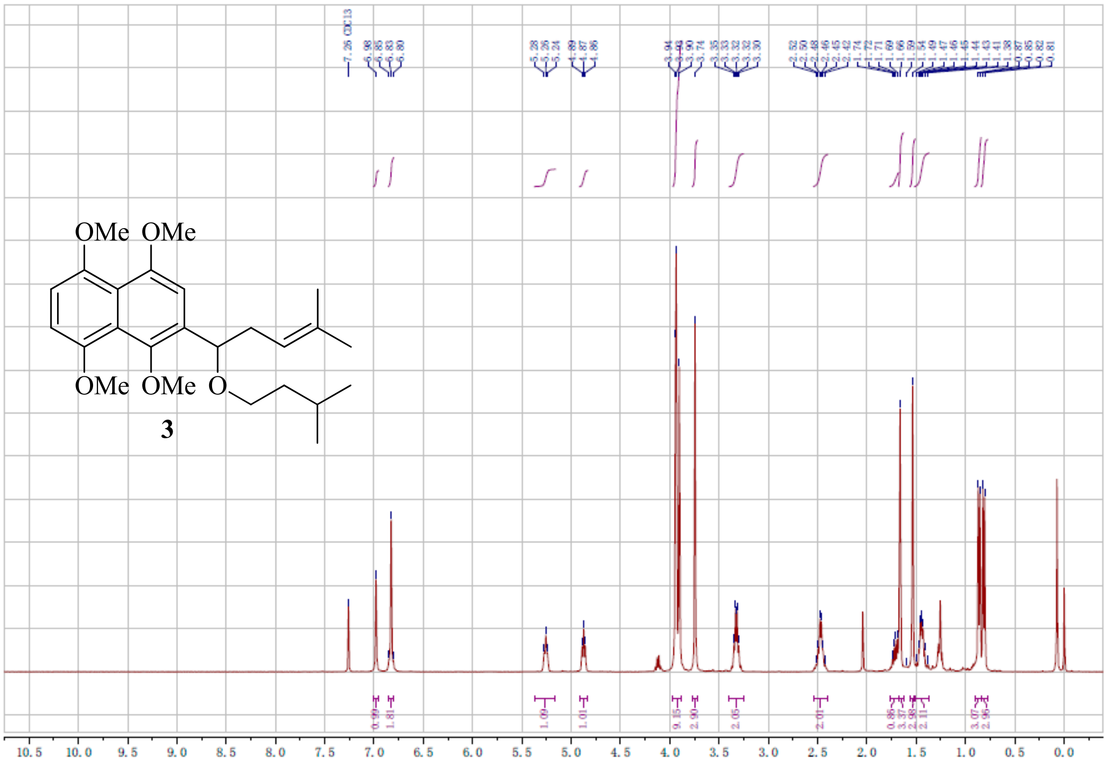

Figure S2. ^{1}H-NMR Spectrum of **6-(1-(isopentyloxy)-4-methylpent-3-en-1-yl)-5,8-dimethoxynaphthalene-1,4-dione** (**4**)

^{1}H-NMR ($CDCl_3$)

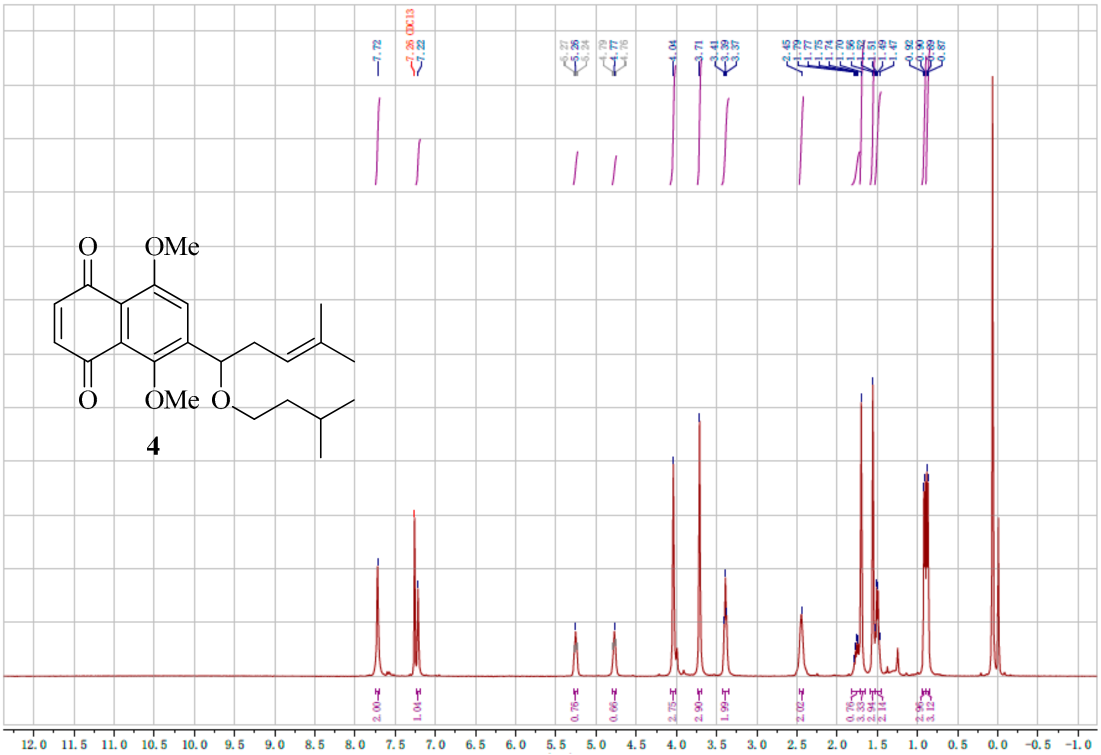

Figure S3. ^{1}H & ^{13}C-NMR Spectra of **(*1E,4E*)-6-(1-(isopentyloxy)-4-methylpent-3-en-1-yl)-5,8-dimethoxynaphthalene-1,4-dione dioxime (5)**

^{1}H-NMR (DMSO-*d*6)

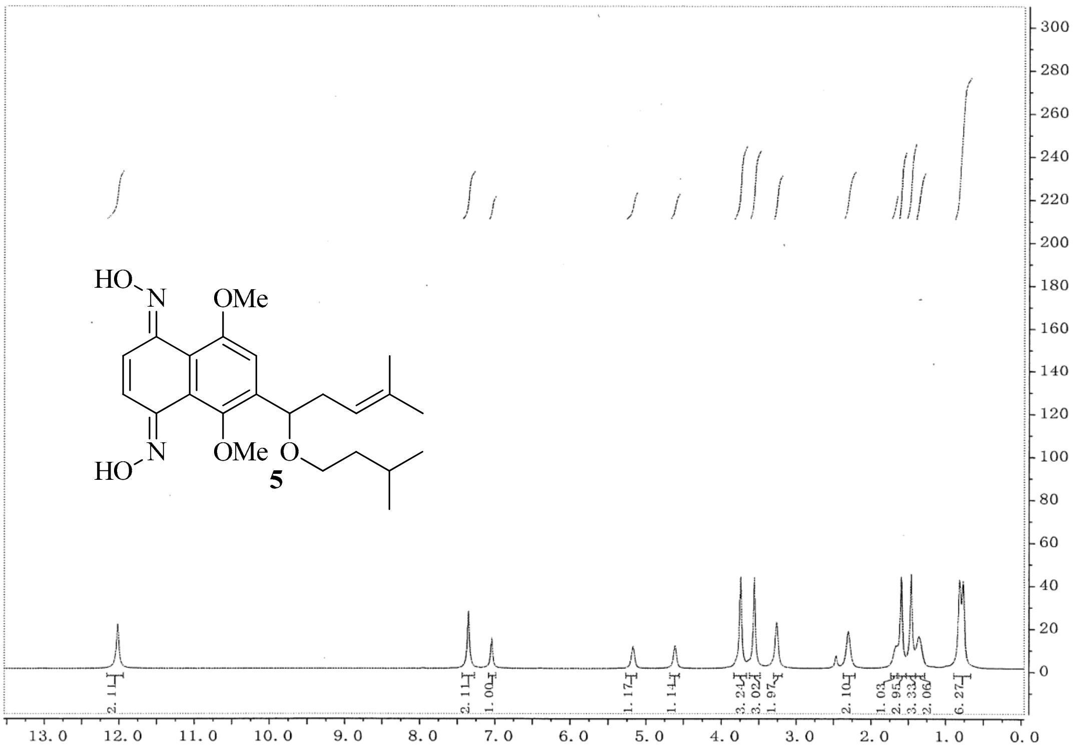

^{13}C-NMR (DMSO-*d*6)

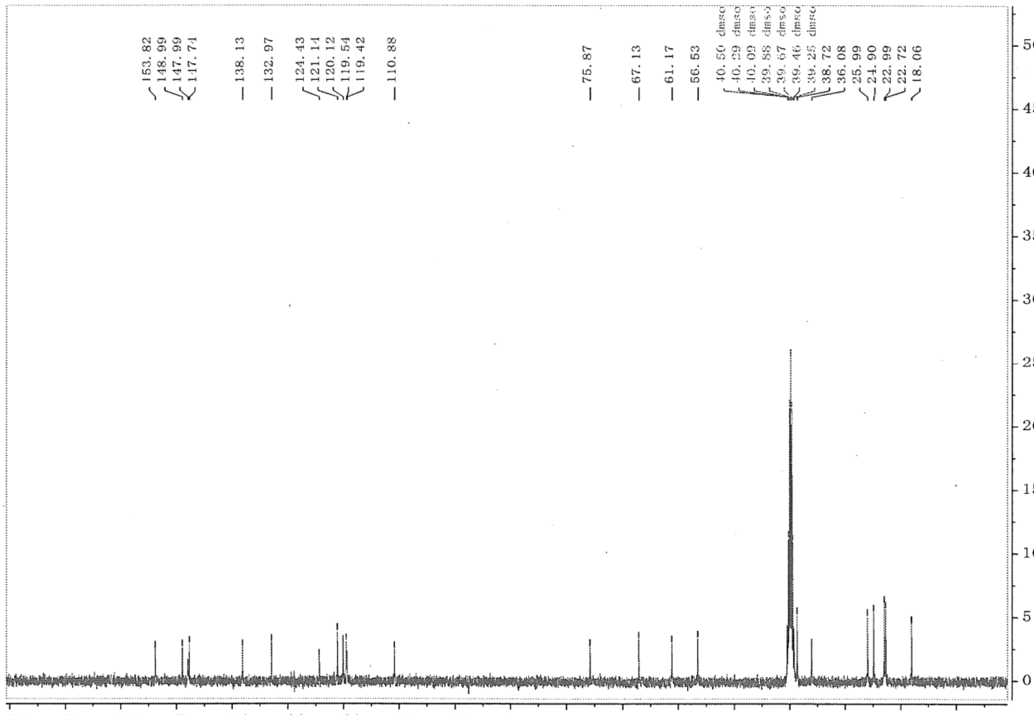

Figure S4. ^{1}H-NMR Spectra of **(*1E,4E*)-6-((*R*)-1-(isopentyloxy)-4-methylpent-3-en-1-yl)-5,8-dimethoxynaphthalene-1,4-dione dioxime** (**14**)

^{1}H-NMR (DMSO-d_6)

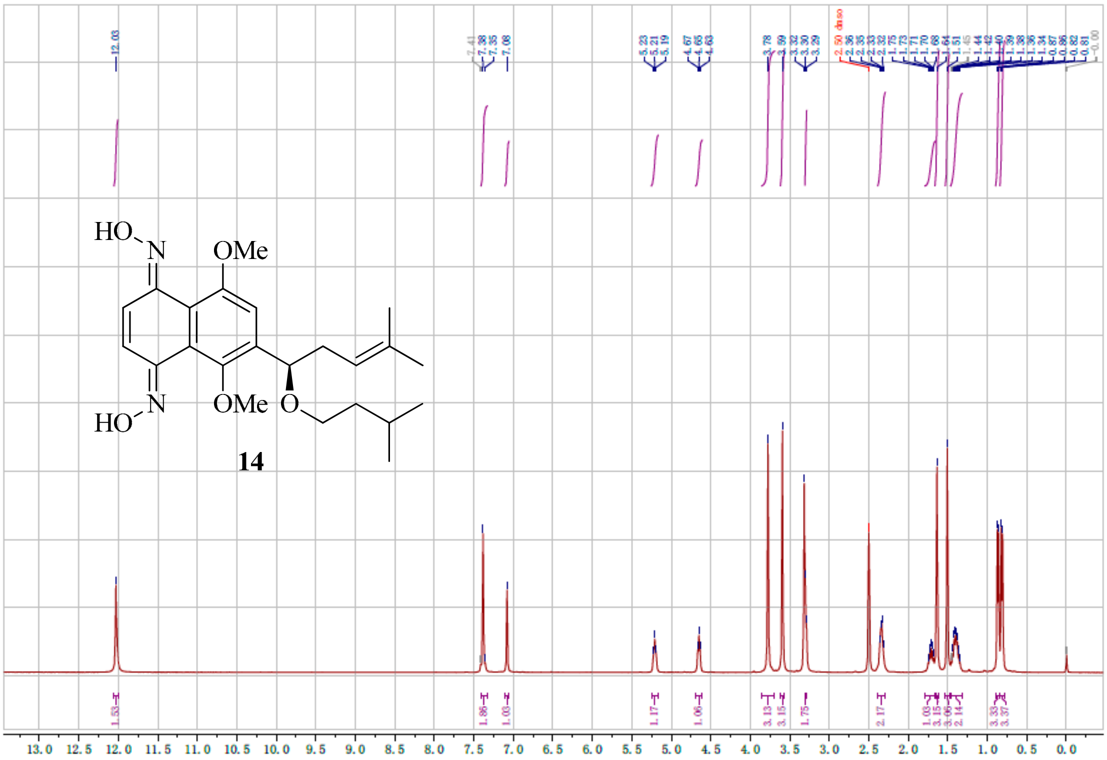

Figure S5. ^{1}H-NMR Spectra of ***(1E,4E)*-6-((*S*)-1-(isopentyloxy)-4-methylpent-3-en-1-yl)-5,8-dimethoxynaphthalene-1,4-dione dioxime** (**15**)

^{1}H-NMR ($CDCl_3$)

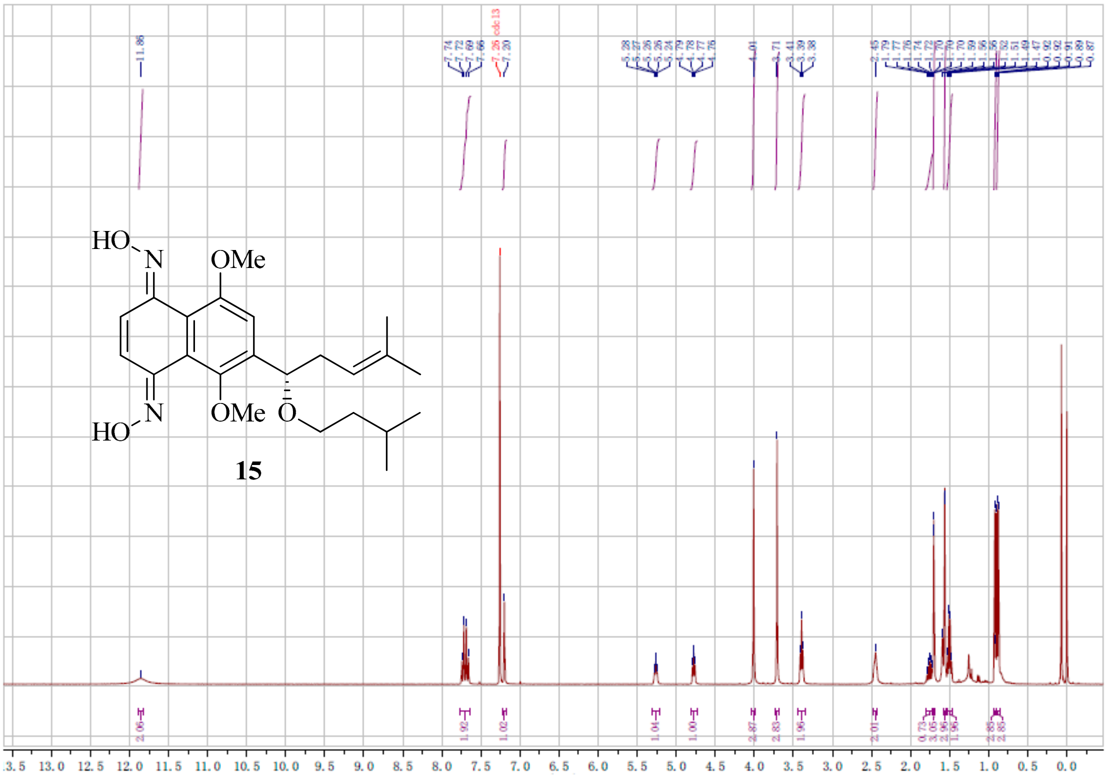

Figure S6. ^{1}H & ^{13}C-NMR Spectra of **(*1E,4E*)-6-(1-((5-hydroxyhexyl)oxy)-4-methylpent-3-en-1-yl)-5,8-dimethoxynaph thalene-1,4-dione dioxime (9)**

^{1}H-NMR (DMSO-d_6)

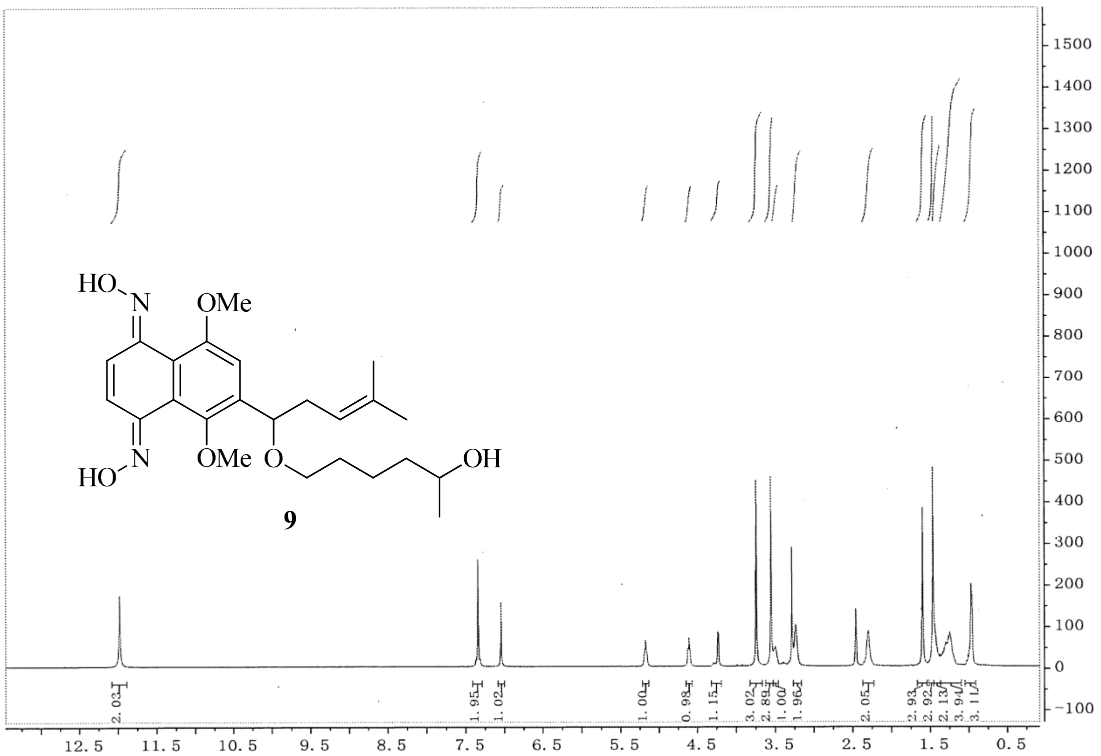

^{13}C-NMR (DMSO-d_6)

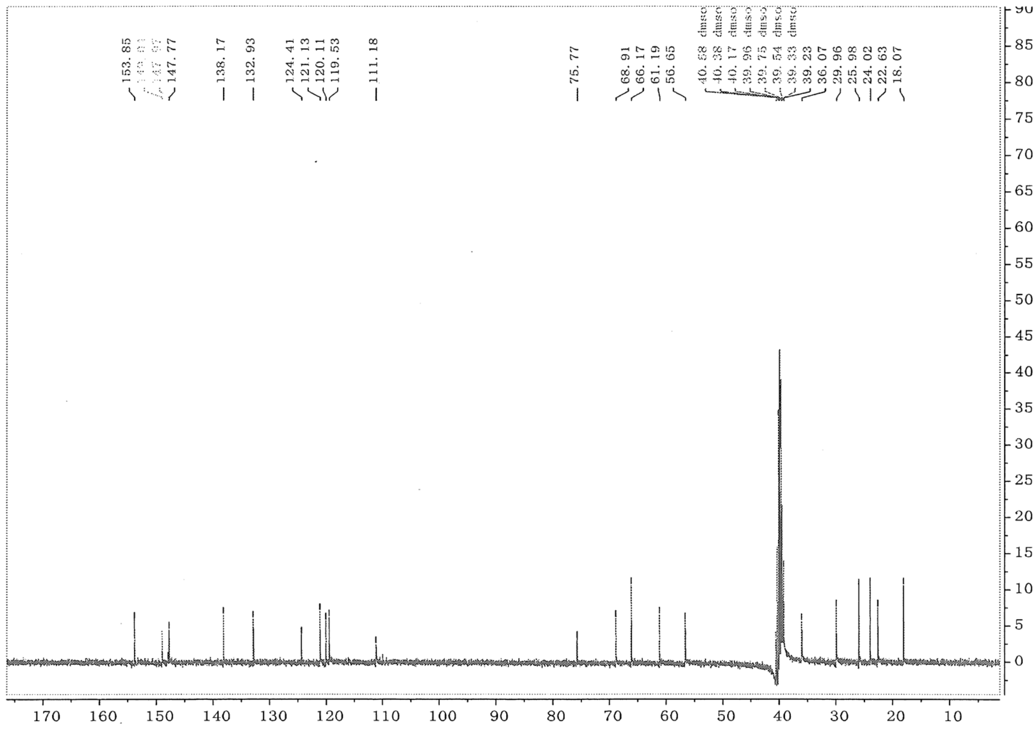

Figure S7. ^{1}H & ^{13}C-NMR Spectra of **(*1E,4E*)-6-(1-((8-hydroxyoctyl)oxy)-4-methylpent-3-en-1-yl)-5,8-dimethoxynaph thalene-1,4-dione dioxime** (**10**)

^{1}H-NMR (DMSO-d_6)

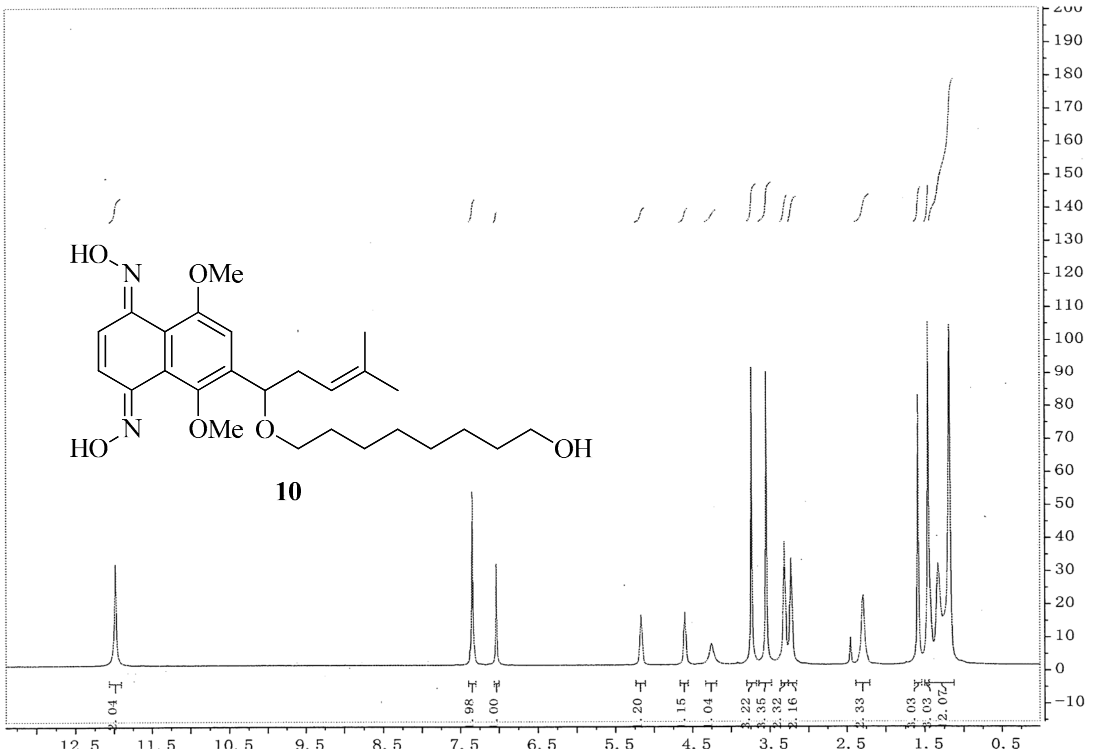

^{13}C-NMR (DMSO-d_6)

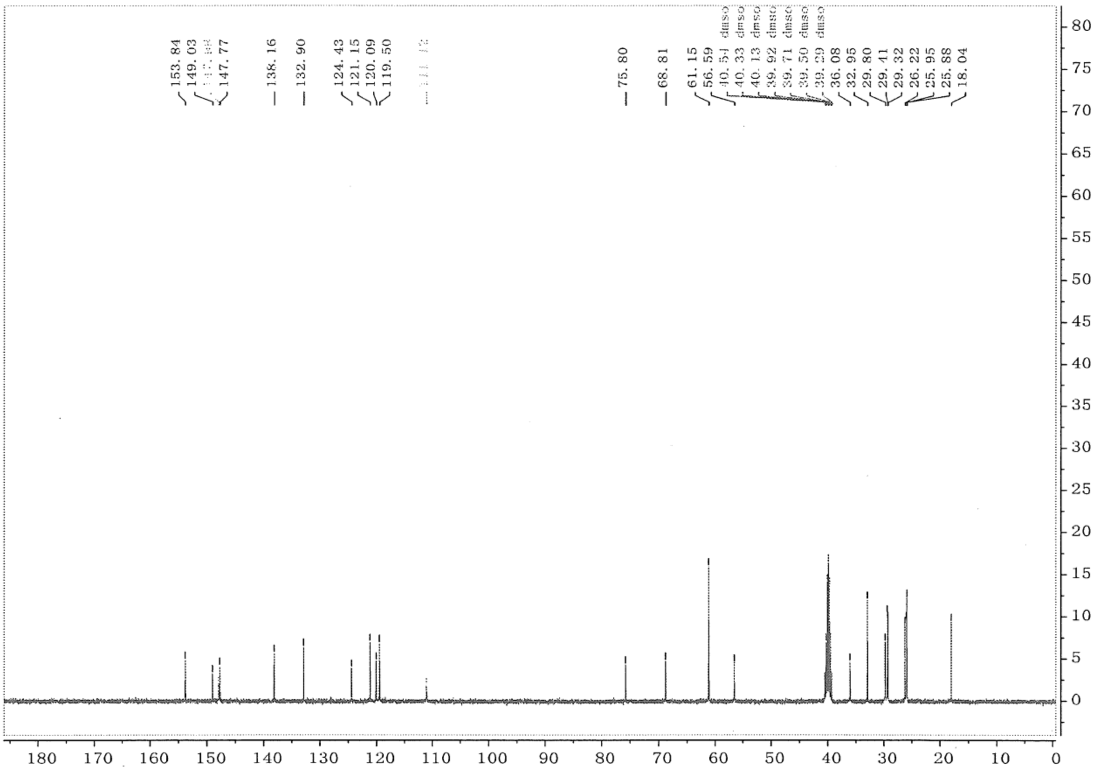

Figure S8. ^{1}H & ^{13}C-NMR Spectra of **(*1E,4E*)-6-(1-(isopentyloxy)-4-methylpent-3-en-1-yl)-5,8-dimethoxynaphthalene-1,4-dione *O,O*-dibenzoyl dioxime (6)**

^{1}H-NMR (CDCl$_3$)

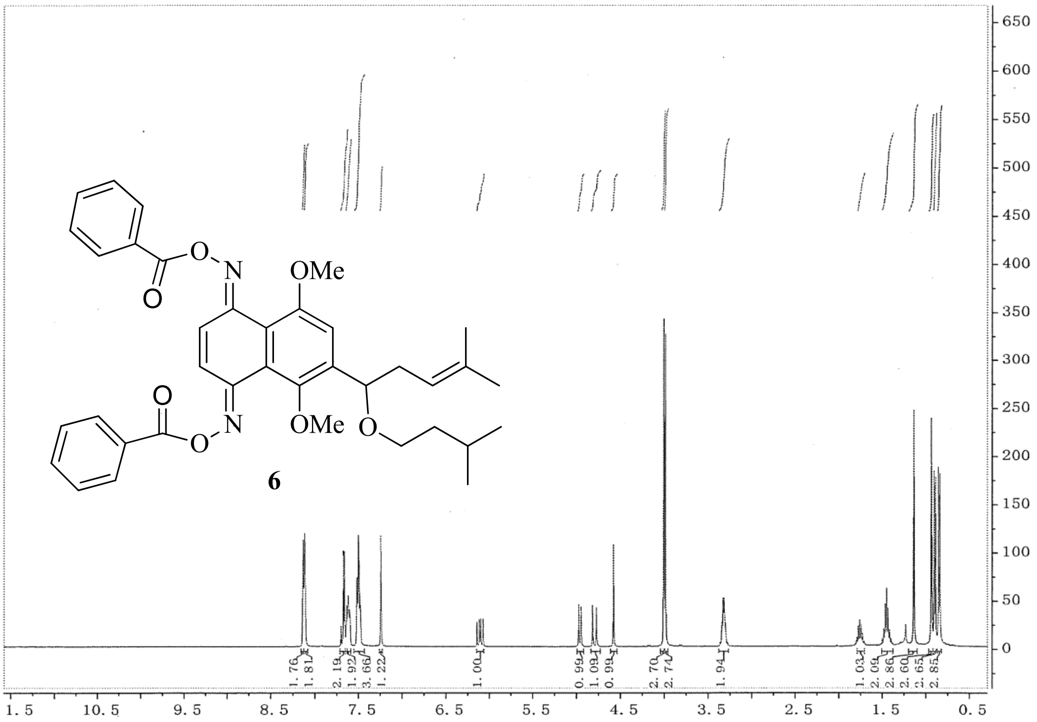

^{13}C-NMR (CDCl$_3$)

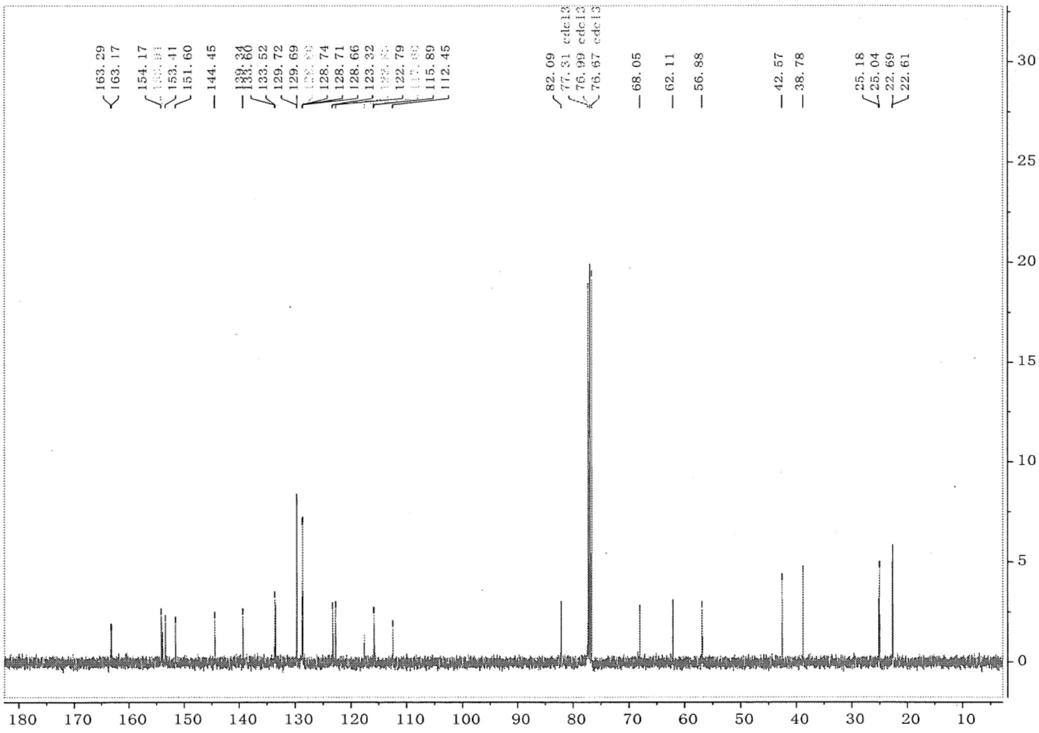

Figure S9. ^{1}H-NMR & ^{13}C-NMR Spectra of **1-((*5E*,*8E*)-5,8-bis(hydroxyimino)-1,4-dimethoxy-5,8-dihydronaphthalen-2-yl)-4-methylpent-3-en-1-yl cinnamate (12)**

^{1}H-NMR (DMSO-d_6)

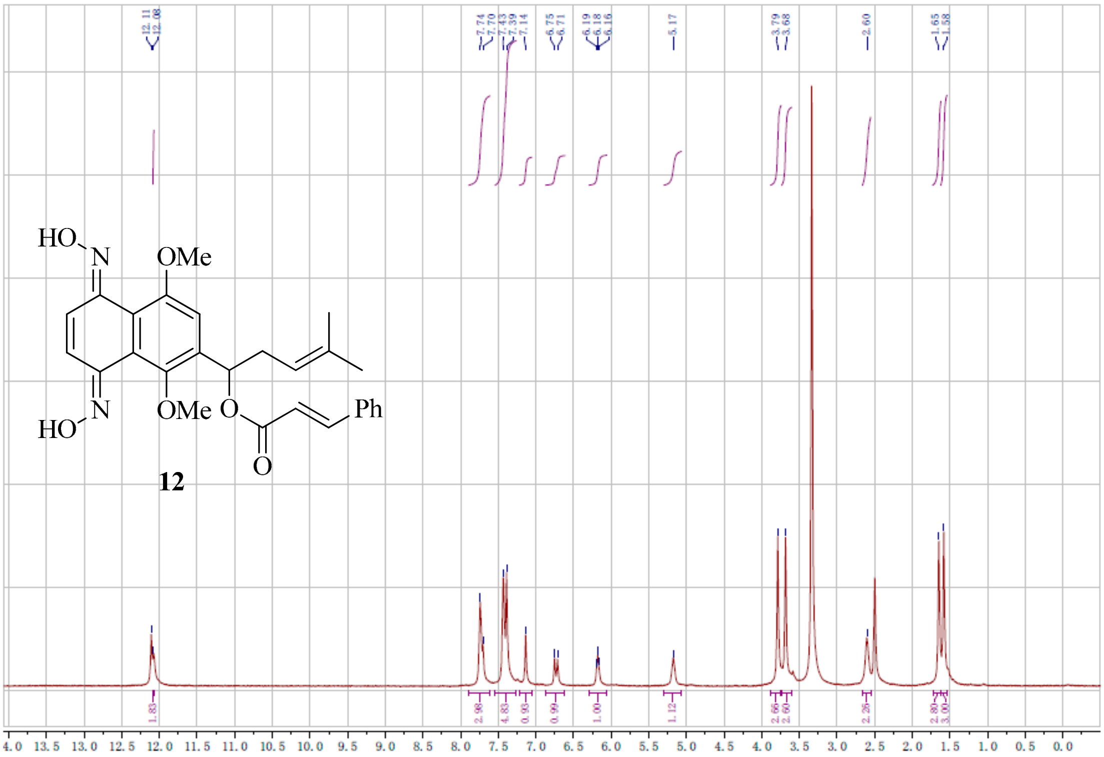

^{13}C-NMR (DMSO-d_6)

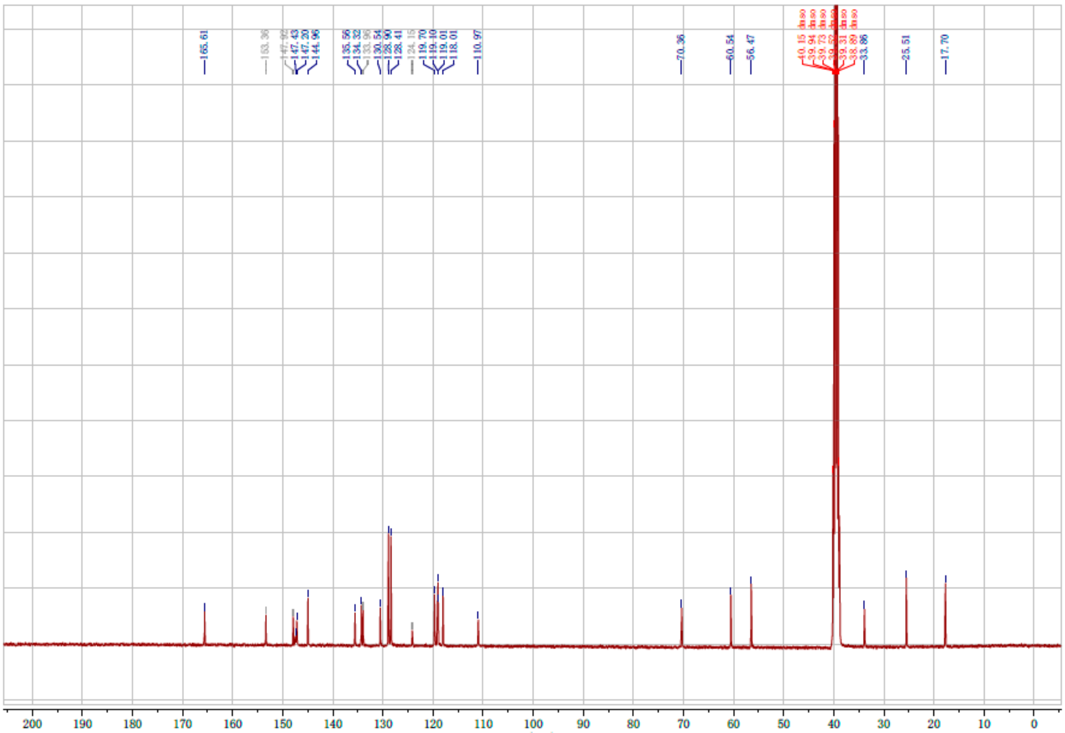

Figure S10. ^{1}H-NMR Spectrum of **(*R*)-4-methyl-1-(1,4,5,8-tetramethoxy naphthalen-2-yl)pent-3-en-1-ol (2-*R*)**

^{1}H-NMR ($CDCl_3$)

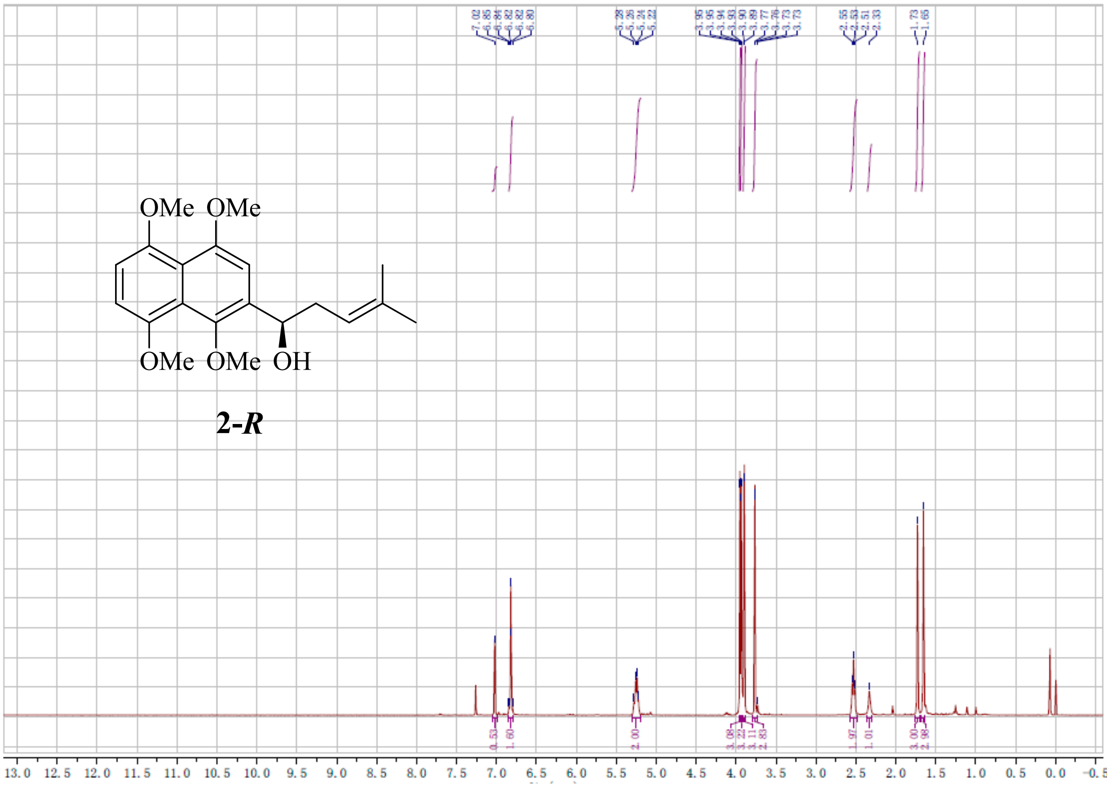

Figure S11. ^{1}H-NMR Spectrum of **(*S*)-4-methyl-1-(1,4,5,8-tetramethoxy naphthalen-2-yl)pent-3-en-1-ol (2-*S*)**

^{1}H-NMR ($CDCl_3$)

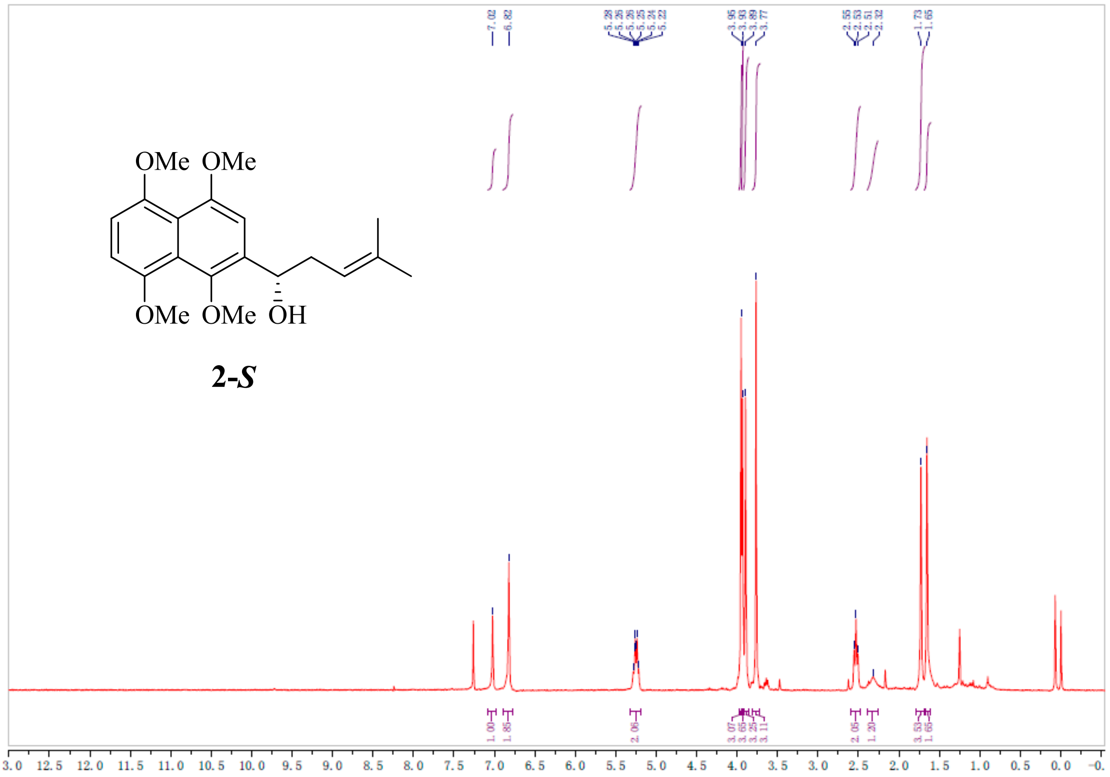

HPLC trace for chiral separation of 2-*R* and 2-*S* as the key intermediates

Chromatographic conditions:

Sino-chiral OD-H column (no. 0A02014-C) with hexane/iPrOH (6:4, V/V) as the eluent, flow rate = 0.8 mL/min, λ = 254 nm.

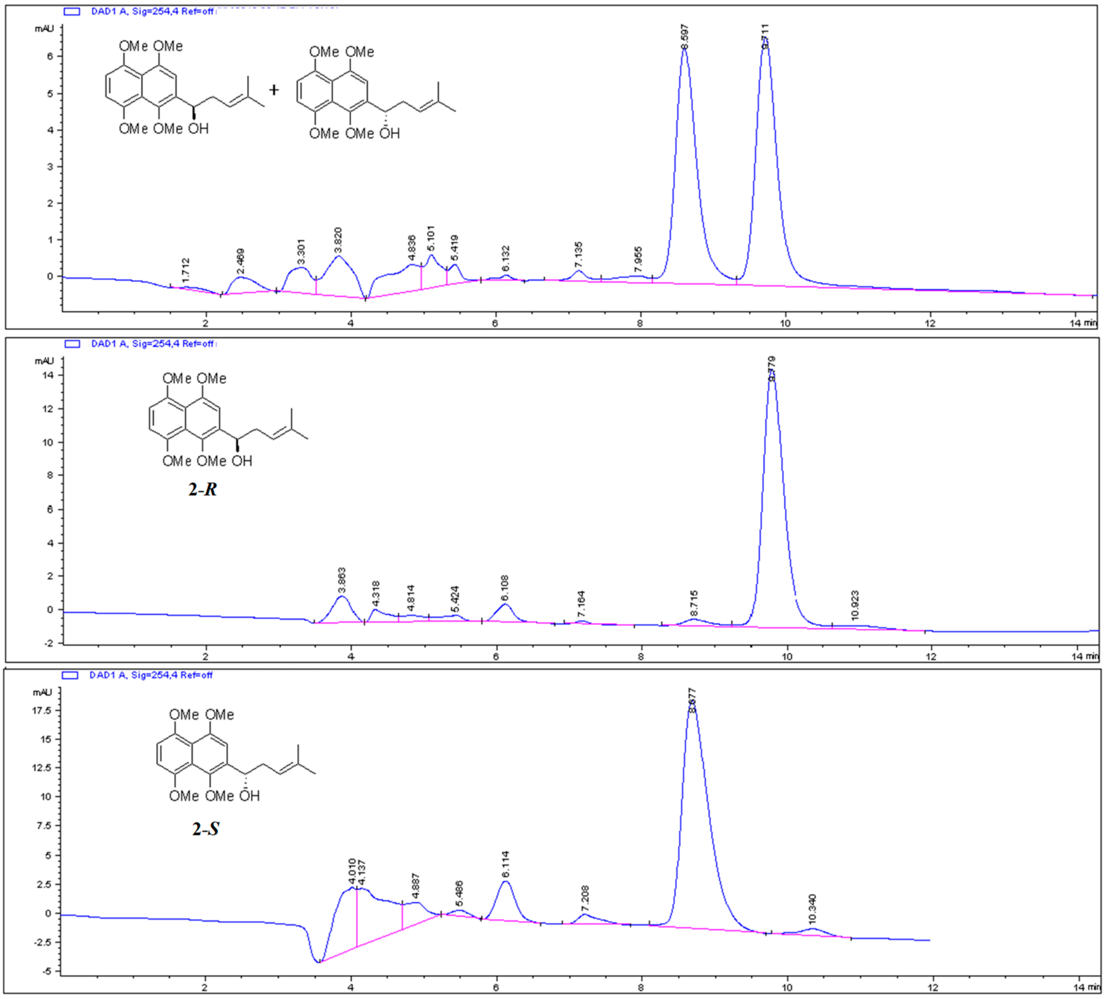

Analytical results:

For compound **2-*R***, which was obtained by hydrogenation of ketone **13** using (+)-DIP-Cl, the retention time is 9.779 min. The enantiomeric excess for compound **2-*R*** is 96% calculated by the normalization method. The retention time for chiral alcohol **2-*S*** is 8.677 min and the enantiomeric excess for this compound is 95%.

HPLC trace for compound 15

Chromatographic conditions:

Agilent ZORBAX C18 column with MeOH/H_2O (3:1, V/V) as the eluent, flow rate = 1.0 mL/min, λ = 318 nm.

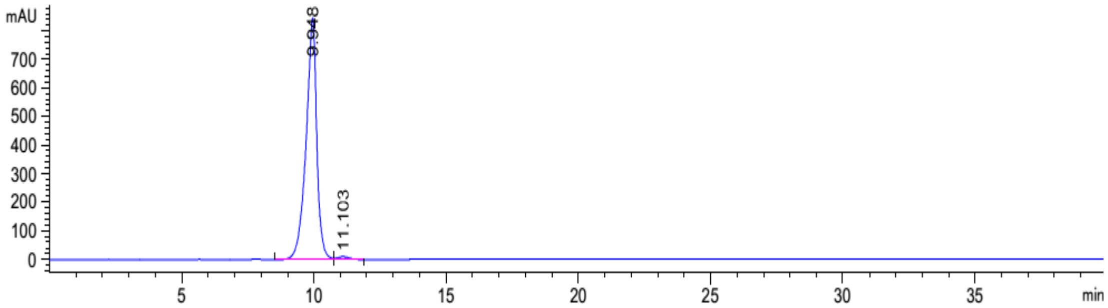

Analytical results:

For compound **2-*R***, which was obtained by hydrogenation of ketone **13** using (+)-DIP-Cl, the retention time is 9.779 min. The enantiomeric excess for compound **2-*R*** is 96% calculated by the normalization method. The retention time for chiral alcohol **2-*S*** is 8.677 min and the enantiomeric excess for this compound is 95%.
